# Phylogenetic Diversity of Nitrogenase Reductase Genes and Possible Nitrogen-Fixing Bacteria in Thermophilic Chemosynthetic Microbial Communities in Nakabusa Hot Springs

**DOI:** 10.1264/jsme2.ME18030

**Published:** 2018-11-07

**Authors:** Arisa Nishihara, Vera Thiel, Katsumi Matsuura, Shawn E. McGlynn, Shin Haruta

**Affiliations:** 1 Department of Biological Sciences, Tokyo Metropolitan University Minami-Osawa, Hachioji, Tokyo 192–0397 Japan; 2 Earth-Life Science Institute, Tokyo Institute of Technology Ookayama, Meguro-ku, Tokyo 152–8551 Japan; 3 Biofunctional Catalyst Research Team, RIKEN Center for Sustainable Resource Science Wako-shi 351–0198 Japan; 4 Blue Marble Space Institute of Science Seattle, WA 98145–1561 USA

**Keywords:** nitrogen fixation, thermophilic bacteria, chemosynthetic communities, geothermal springs, *nifH* gene

## Abstract

Chemosynthetic microbial communities develop and form dense cell aggregates in slightly alkaline sulfidic hot springs in the temperature range of 70–86°C at Nakabusa, Japan. Nitrogenase activity has recently been detected in the microbial communities collected. To identify possible members capable of nitrogen fixation, we examined the diversities of 16S rRNA and nitrogenase reductase (NifH) gene sequences in four types of chemosynthetic communities with visually different colors and thicknesses. The results of a 16S rRNA gene analysis indicated that all four microbial communities had similar bacterial constituents; the phylum *Aquificae* was the dominant member, followed in abundance by *Thermodesulfobacteria*, *Firmicutes*, and *Thermotogae*. Most of the NifH sequences were related to sequences reported in hydrothermal vents and terrestrial hot springs. The results of a phylogenetic analysis of NifH sequences revealed diversity in this gene among the communities collected, distributed within 7 phylogenetic groups. NifH sequences affiliated with *Aquificae* (*Hydrogenobacter*/*Thermocrinis*) and *Firmicutes* (*Caldicellulosiruptor*) were abundant. At least two different energy metabolic pathways appeared to be related to nitrogen fixation in the communities analyzed; aerobic sulfur/hydrogen-oxidizing bacteria in *Aquificae* and fermentative bacteria in *Firmicutes*. The metabolic characteristics of these two dominant phyla differed from those previously inferred from nitrogenase activity assays on chemosynthetic communities, which were associated with hydrogen-dependent autotrophic sulfate reduction. These assays may correspond to the observed NifH sequences that are distantly related to the known species of *Thermodesulfovibrio* sp. (*Nitrospirae*) detected in the present study. The activities of nitrogen-fixing organisms in communities may depend on redox states as well as the availability of electron donors, acceptors, and carbon sources.

Biological nitrogen fixation supplies nitrogen to ecosystems by reducing N_2_ to NH_3_, which may be incorporated into the biomass. This process accounts for the majority of nitrogen transferred from the atmospheric reservoir into the biosphere. Nitrogen fixation is a significant factor influencing the development of macro- and microbial communities, particularly in environments in which the supply of nitrogen compounds is less than that of carbon, *e.g.*, photosynthesis-driven (76), chemolithotroph-dominated (35, 49, 55), and plant-associated (53, 57, 60, 75) microbial communities and gut microflora in wood-feeding termites (52).

Biological nitrogen fixation is catalyzed by the nitrogenase enzyme system (6) and requires additional protein maturation factors encoded by *nif* genes (4, 28). The complex metalloenzyme active site of nitrogenase, which is harbored by the proteins encoded by *nifDK* genes, receives electrons from the nitrogenase reductase protein encoded by *nifH*. Nitrogenase reductase (NifH) binds NifDK, delivers electrons, and hydrolyzes two ATP per electron transferred to NifDK (17, 61, 71). Since nitrogenase reductase exhibits high sequence similarity among various nitrogen fixers (27), *nifH* has been used as a marker gene for the detection of possible nitrogen-fixing microbes in different environments (14, 24, 78).

Accumulated sequence information on nitrogenase and nitrogenase reductase may be useful for testing hypotheses on the evolutionary history of nitrogenase and the physiology of nitrogenase-harboring organisms through time (3, 4). A detailed knowledge of thermophilic nitrogen-fixing microbes may provide valuable information for the study of the early evolution of organisms, particularly because thermophilic chemosynthetic microbial communities in terrestrial hot springs and deep-sea hydrothermal vents may resemble ancient biological communities before the appearance of photosynthesis on the earth (15, 64).

Thermophilic nitrogen fixation has been reported in some bacterial communities in terrestrial hot springs at ≤63.4°C, which include the unicellular cyanobacterium *Synechococcus* and filamentous heterocystous cyanobacterium *Mastigocladus* (1, 65, 77). In methanogenic archaea, *Methanocaldococcus* sp. FS406-22 isolated from deep-sea hydrothermal vent fluid exhibited nitrogenase activity up to 92°C (42). Previous studies on *nifH* genes in hydrothermal vents suggested the existence of uncultured thermophilic nitrogen-fixing bacteria and archaea (7, 18, 42, 50, 63). In studies on terrestrial hot springs in Yellowstone National Park, *nifH* genes were detected in sediments under hot spring waters at acidic and neutral pH at 76–89°C (20, 21, 39). These studies documented the distribution of nitrogen-fixing microbes in various bacterial and archaeal lineages in thermal environments. However, a more comprehensive understanding of the nitrogen-fixing activities of thermophiles has been hampered by a shortage of isolates from thermal environments as well as *in situ* biological data.

In hot springs at Nakabusa, Japan, densely packed chemosynthetic microbial communities—so-called microbial mats and streamers—develop in slightly alkaline and sulfidic hot spring water at 70–86°C (11, 47, 48, 67). The chemical composition of hot spring water remains stable over yearly time scales; pH is slightly alkaline (pH 8.5–8.9) and the water contains approximately 0.10–0.25 mmol L^−1^ sulfide, 0.019–0.246 mmol L^−1^ sulfate, and 5.0–6.1 μmol L^−1^ ammonia (30, 32, 47, 48). Nitrate and nitrite were not detected (30, 33, 48). Uncultured chemosynthetic bacteria-dominated mats exhibited high *in situ* biomass production, with even greater CO_2_ uptake than photosynthetic cyanobacterial mats at 70°C in Yellowstone National Park (32). The findings of 16S rRNA gene sequence-based community analyses of the chemosynthetic mats and streamers at Nakabusa were initially reported by Nakagawa and Fukui (47). These communities contained a high abundance of uncultured chemolithoautotrophic *Sulfurihydrogenibium* members in the phylum *Aquificae*, which are often referred to as ‘large sausage-shaped bacteria’ because of their morphology and long length (5–40 μm) (11, 47, 48, 67). The uncultured bacteria aerobically oxidize sulfide, which is continuously supplied from the hot spring water, and actively fix carbon dioxide (67, 68).

We recently detected and characterized the nitrogenase activity of thermophilic chemosynthetic microbial communities in Nakabusa hot springs (49). This activity required sulfate, H_2_, and CO_2_, which suggested the participation of chemolithoautotrophic sulfate-reducing metabolism (49). In the present study, we analyzed the diversity of *nifH* and 16S rRNA gene sequences in these microbial communities in order to identify the candidates responsible for the nitrogenase activity reported previously (49). Different colored (pale-tan, white, and gray) chemosynthetic microbial communities in the forms of mats and streamers at 72–77°C were comparatively analyzed.

## Materials and Methods

### Sample collection and geochemical features of hot springs

**Fig. 1:**
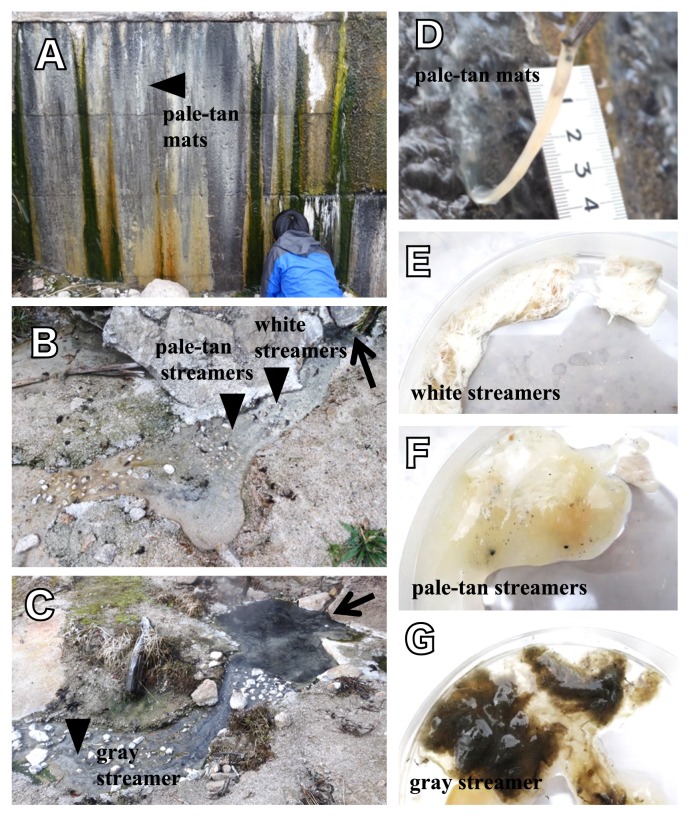
Photo images of sampling sites, microbial mats, and streamers. **A**, Microbial mats on the concrete wall covered with hot spring water at the Wall Site; **B**, **C**, Hot spring water streams at the Streamer Site. Pointed arrows show sources of hot spring water; **D**, Typical pale-tan colored microbial mats at the Wall Site; **E**, **F**, Typical white streamers and pale-tan streamers collected in 9-cm Petri dishes from the Streamer Site shown in B. These streamers were found in the stream at a depth <2 cm; **G**, Typical gray streamers in a Petri dish from the stream at a depth <10 cm shown in C. In **A** and **D**, the same photo images were used as those in our previous study (49).

Microbial communities that developed in a temperature range between 72 and 77°C were collected from two sites, *i.e.*, Wall Site [36°23′20″N, 137°44′52″E] (Fig. 1A) and Stream Site [36°23′33″N, 137°44′52″E] (Fig. 1B and C), on 22 July 2016 at Nakabusa hot springs located in Nagano prefecture, Japan. The electric conductivity (EC) and oxidation-reduction potential (ORP) of the hot spring water were 45–55 mS m^−1^ and −280 to −215 mV, respectively. A Ag/AgCl_2_ reference electrode was used to measure ORP.

Pale-tan microbial communities in the form of mats were collected at the Wall Site (Pale-tan mats, Fig. 1A and D). The pale-tan mat had developed at 75°C on the concrete wall at a water depth of less than 1 cm and water current velocity of 40 cm s^−1^ at the surface (Fig. 1A). At the Stream Site, three types of microbial communities in the form of streamers were collected; whitish (white streamers, 77°C) (Fig. 1B and E), grayish (gray streamers, 75°C) (Fig. 1C and G), and pale-tan (pale-tan streamers, 72°C) (Fig. 1B and F). White and paletan streamers developed at a water depth of less than 2 cm in a stream with current velocities of 60 cm s^−1^ and 15 cm s^−1^, respectively (Fig. 1B). Gray streamers developed at a deeper depth (up to 10 cm) with a medium current velocity (30 cm s^−1^) (Fig. 1C and G) and had a stickier appearance.

For DNA analyses, samples were placed in 2.0-mL screw cap plastic tubes, frozen immediately in a dry ice-ethanol slurry on site, stored on dry ice for several hours during transport, and then placed in a freezer at −80°C until used, as described previously (49).

### DNA extraction and 16S rRNA gene and *nifH* gene amplicon analyses

DNA extraction from the mats and streamers as well as 16S rRNA gene sequence analyses were performed as described previously (49). Briefly, bulk DNA was isolated from approximately 100 mg (wet weight) of the sample using bead beating and a chloroformphenol extraction protocol (51), combined with the cetyltrimethylammonium bromide (CTAB) method. Regarding pale-tan mats and streamers, DNA extracts used for 16S rRNA gene amplicon analyses in our previous study (49) were employed for *nifH* gene amplicon analyses in the present study.

PCR was performed to amplify the V4 region of 16S rRNA genes using the primers 515F and 806R (9). *nifH* gene fragments were amplified with two different primer sets: MehtaF (5′ GGHAARGG HGGHATHGGNAARTC 3′) and MehtaR (5′ GGCATNGCRAAN CCVCCRCANAC 3′) as well as PolF (5′ TGCGAYCCSAARGC BGACTC 3′) and PolR (5′ ATSGCCATCATYTCRCCGGA 3′) (42, 56). The PolF/PolR set has been widely applied in microbial community analyses of moderate temperature environments (13, 45, 56); however, its coverage efficiency was reported to be low by an *in silico* analysis (13). The other primer set, MehtaF/MehtaR, was designed to cover bacterial and archaeal *nifH* genes, and has been used in studies of thermal environments, such as hydrothermal vents and hot springs in Yellowstone National Park (39, 41). The expected fragment size amplified with PolF/PolR and MehtaF/MehtaR was 362 bp covering positions 115–476 and 389 bp covering positions 28–416, respectively, in the *Azotobacter vinelandii* reference sequence of *nifH* (M20568) (13). In comparisons of coverage between the two primer sets, the MehtaF/MehtaR primer set widely covers known *nifH* sequences, while the PolF/PolR primer set additionally covers unique sequences that are not covered by the MehtaF/MehtaR primer set based on the findings of an *in silico* survey against the *nifH* database by Heller *et al.* in the ARB program (24, 40), as shown in Table S1, S2, and S3. PCR for *nifH* gene amplification was performed in 50 μL total volume reaction mixtures, containing 2.5 mmol L^−1^ of MgCl_2_, 5 μL of 10×*Ex Taq* Buffer (Mg^2+^-free) (Takara, Shiga, Japan), dNTP Mixture (0.2 mmol L^−1^ each), 1 μmol L^−1^ primers, 1.2 units of *Ex Taq* DNA polymerase (Takara), and 14 to 82 ng of extracted DNA. The amplification reaction started with initialization at 94°C for 2 min, followed by 35 cycles of denaturation at 94°C for 1 min, annealing at 55°C for 1 min, and elongation at 72°C for 2 min. Amplified products were visualized on ethidium bromide-stained 2% (w/v) agarose gels with Gene Ladder 100 (0.1–2 kbp, Nippon Gene, Tokyo, Japan) as DNA size markers and purified using the QIAGEN II Gel Extraction Kit (Qiagen, Hilden, Germany). Duplicate PCR products were pooled and quantified with the dsDNA Broad Range assay on a Qubit fluorometer (Life Technologies, Grand Island, NY, USA). DNA fragments were subjected to paired-end sequencing using an Illumina MiSeq platform (Illumina, San Diego, CA, USA) at Fasmac (Atsugi, Japan). The sequences of the 16S rRNA gene and *nifH* gene were quality filtered and analyzed with the Quantitative Insights Into Microbial Ecology (QIIME) pipeline (version 1.9.0 [8]). The sequences of the 16S rRNA gene and *nifH* gene were quality filtered with sickle tools based on a quality score cut-off of 20 and pair-end sequences with a length of more than 130 bp were selected for analysis. Chimera checks were performed using USEARCH and the Greengenes 16S rRNA database, and then clustered into OTUs with ≥97% nucleotide sequence identity in QIIME by *de novo* OTU picking. The sequences of the *nifH* gene were clustered into OTUs with ≥97% nucleotide sequence identity using USEARCH (10). The OTUs of the *nifH* gene were generated from the combined datasets of sequences amplified by both primer sets. 16S rRNA amplicon sequence data of the pale-tan mats and streamers analyzed in our previous study were used after removing singleton sequences (BioSample accession numbers, SAMD00089769–SAMD00089770 [SSUB008209]) (49). After removing singleton OTUs, close relatives of representative OTUs were identified by a BLASTn (for 16S rRNA genes) or BLASTX (for NifH) analysis. A phylogenetic analysis for the 16S rRNA gene was conducted using the SILVA database (SILVA SSU Ref NR_128 database) and the backbone tree (tree_SSURefNR99_1200slv_128) in the ARB program (40). *nifH* sequences were translated into amino acid sequences using MEGA7 (37). The primers used in the present study precluded a full-length sequence analysis; however, one required residue, Cys 97 (protein numbering for NifH in *A. vinelandii*) was confirmed in the sequences; Cys 97 and Cys 132 are the 4Fe-4S iron sulfur cluster ligating cysteines (26). The putative NifH sequences amplified by the PolF/PolR and MehtaF/MehtaR primer sets were used in the phylogenetic analysis, including nucleotide sequences containing stop codons. Amino acid sequences were aligned using ClustalW with the default settings implemented in MEGA7, a phylogenetic tree was constructed using Maximum Likelihood with the WAG model for substitutions with 100 bootstrap replicates, and an estimated Gamma distribution was used to model evolutionary rate differences among sites in the ARB program (37, 40). The proportion of invariable sites was estimated using four categories of substitution rates. We used 310 positions in the final dataset to construct a phylogenetic tree. Sequences of the *nifH* gene homologous to *Methanococcus voltae* (CAA27407), *Methanocaldococcus fervens* AG86 (ZP 04795296), *M. vulcanius* M7 (ACX72742), and *Methanothermococcus okinawensis* H1 (EFL49005) were used as the outgroup.

### Nucleotide sequence accession numbers

Sequences were deposited in DDBJ/EMBL/GenBank under BioProject accession number PRJDB6157 (PSUB007578) and BioSample accession numbers SAMD00112298–SAMD00112299 (SSUB009105) for the 16S rRNA gene sequences of white and gray streamers and BioProject accession number PRJDB6756 (PSUB008319) and BioSample accession numbers SAMD00089769–SAMD00112299 (SSUB008209, SSUB009105) for the *nifH* gene sequences of four samples. The representative *nifH* sequences shown in Fig. 3 and Table S6 and the 16S rRNA gene sequences listed in Table S5 were deposited in DDBJ/EMBL/GenBank with accession numbers from LC380447 to LC380501 for *nifH* and LC381388 to LC381416 for 16S rRNA.

## Results and Discussion

### Microbial community analysis based on 16S rRNA genes

Between 29,671 to 44,028 reads were obtained by amplification from the four samples, corresponding to a range of OTU’s from 529 to 642 OTUs that were obtained for the 16S rRNA genes from four samples collected in this study and a previous study (Table S4) (49). Twenty-nine OTUs with ≥0.2% relative abundance in at least one sample (29 OTUs) are listed in Table S5.

**Fig. 2:**
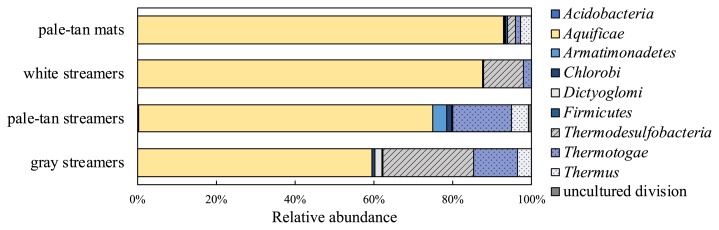
Microbial community analysis based on the 16S rRNA gene. Relative abundance is shown at the phylum level. 16S rRNA amplicon sequence data of the pale-tan mats and streamers analyzed in our previous study were used after treatments removing singleton sequences (49). OTUs with >0.2% relative abundance in at least one community were used for the figure.

The relative abundance of sequences in each sample is shown at the phylum level in Fig. 2. Based on the 16S rRNA sequence analysis, bacteria of the phylum *Aquificae* were the most dominant constituents in all samples (51.1 to 76.1% of the total reads). In this phylum, three genera were detected: *Sulfurihydrogenibium*, *Hydrogenobacter*, and *Thermocrinis*. 16S-OTUs related to *Sulfurihydrogenibium* were the most abundant in all samples; pale-tan mats (72.9% of the total reads), white streamers (78.9% of the total reads), pale-tan streamers (54.8% of the total reads), and gray streamers (23.1% of the total reads) (Table S5). The most abundant 16S-OTU, denovo14971, belonging to the genus *Sulfurihydrogenibium* showed 100% identity to *Sulfurihydrogenibium azorense* as well as the uncultured “large sausage-shaped bacteria”, which were previously described as the dominant members of whitish microbial streamers in Nakabusa (67, 68). The sample of gray streamers additionally contained similarly abundant *Thermocrinis* sequences (23.6% of the total reads) (Table S5).

Other phyla detected in the communities by the 16S rRNA gene sequence analysis were *Acidobacteria*, *Armatimonadetes*, *Chlorobi*, *Dictyoglomi*, *Firmicutes*, *Thermodesulfobacteria*, *Thermotogae*, and *Deinococcus-Thermus*, with varying relative abundances (Fig. 2). Two 16S rRNA gene OTUs were detected in the phylum *Thermodesulfobacteria*. 16S-OTU denovo20616, which showed 98.8% identity to the sulfur-reducing bacterium *Caldimicrobium rimae* (44), accounted for 1.20, 8.69, 0.22, and 21.4% of the total reads in pale-tan mats, white streamers, pale-tan streamers, and gray streamers, respectively (Table S5). The other 16S-OTU in the phylum *Thermodesulfobacteria* was 100% identical to *Caldimicrobium thiodismutans*, a sulfur-disproportionating bacterium recently isolated from Nakabusa (34). Other 16S-OTUs found in all communities with relatively high abundance belonged to the genus *Caldicellurosiruptor* in the phylum *Firmicutes* (16S-OTU denovo9761), *Thermus* in the phylum *Deinococcus-Thermus* group (16S-OTU denovo18496), and the genus *Fervidobacterium* in *Thermotogae* (16S-OTU denovo17765). Some of the 16S-OTUs in the phylum *Thermotogae* (16S-OTUs denovo45350, 46213, and 31056), *Chlorobi* (16S-OTUs denovo27638 and denovo54962), and an uncultured division of the BP4 lineage (16S-OTU denovo56209) showed low sequence identities to known isolates (less than 88.4% identity). These uncultured clades in *Thermotoga* (EM3 lineage) and *Chlorobi* (OPB56 lineage) were also reported in photosynthetic microbial communities at 60°C at the siliceous and slightly alkaline Mushroom Spring in Yellowstone National Park (69, 70).

### Distribution and diversity of *nifH* genes

*nifH* gene fragments were amplified by PCR using two different primer sets from all four samples, yielding between 5,655 and 22,248 reads and between 31 and 45 NifH-OTUs in the amplicons obtained by the MehtaF/MehtaR primer set, and between 1,848 and 25,897 reads and between 21 and 71 NifH-OTUs in the amplicons by the PolF/PolR primer set (Table S4).

**Fig. 3:**
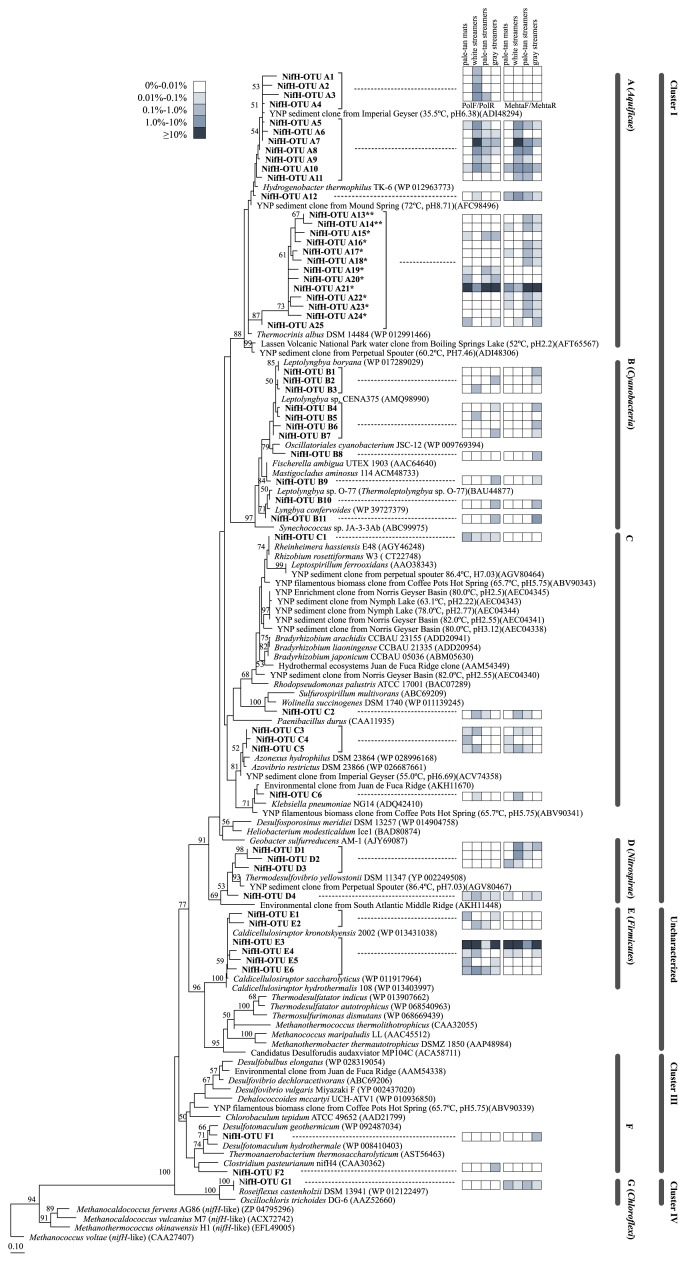
Maximum-likelihood phylogenetic tree of NifH sequences obtained from four microbial communities amplified by two primer sets. Bootstrap values of more than 50% are indicated at the respective nodes. The color of the column on the right-hand of the NifH-OTU name indicates the relative abundance of the NifH-OTUs in each community analyzed with either primer set. The NifH classification of groups was named A to G based on clustering in the phylogenetic tree. When the groups clustered with known sequences from a single phylum, the phylum name was added in parentheses after the group name. NifH clusters defined by Zehr *et al.* are also shown (78). *, NifH-OTUs containing stop codons at the end 15 amino acids of the obtained NifH sequences; **, NifH-OTUs containing stop codons at the end 15 amino acids and in the middle of the obtained NifH sequences.

**Fig. 4:**
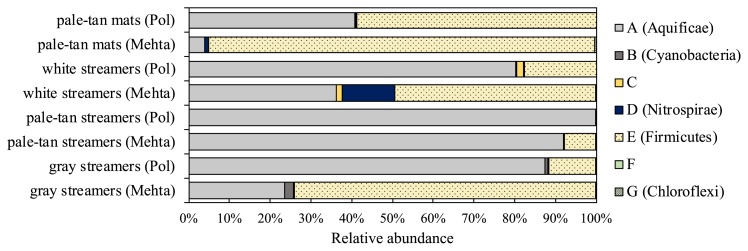
Relative abundance of phylogenetic clusters based on NifH sequences. NifH-OTUs detected by the PolF/PolR or MehtaF/MehtaR primer set with >0.1% abundance in at least one community were used for calculations. The names of communities are followed by the names of the PCR primer sets in parentheses given in the vertical axis; “Pol” for PolF/PolR and “Mehta” for MehtaF/MehtaR.

The phylogenetic tree in Fig. 3 was constructed with 55 NifH-OTUs amplified by the PolF/PolR and MehtaF/MehtaR primer sets and presented >0.1% relative abundance in at least one sample (Table S6). The phylogenetic relationships of NifH amino acid sequences elucidated here with the reported NifH sequences are shown in the maximum-likelihood tree. The NifH-OTUs detected in this study were distributed to seven phylogenetic groups (groups A–G) and four NifH clusters defined by Zehr *et al.* (78) (Fig. 3). As shown in Fig. 4, amplification by PolF/PolR led to a higher relative abundance of group A clustered with sequences from the phylum *Aquificae*, while, PCR products amplified by MehtaF/MehtaR contained more sequences of group E, clustered with reported sequences from *Firmicutes*. No sequence affiliated with known archaeal sequences was detected. Eight NifH-OTUs were detected in all four communities (Fig. 3 and Table S6); NifH-OTU A5, A10, A12, and A21 in group A (*Aquificae*), NifH-OTU C1 in group C, NifH-OTU D4 in group D (*Nitrospirae*), NifH-OTU E3 and E6 in group E (*Firmicutes*), and NifH-OTU G1 in group G (*Chloroflexi*). Fig. 4 shows that NifH-OTUs in groups A (*Aquificae*) and E (*Firmicutes*) were the most abundant overall.

### Possible nitrogen fixers related to chemolithotrophic sulfate-reducing metabolism

We previously showed nitrogenase activity with the acetylene reduction method in pale-tan mats at ≥70°C in Nakabusa hot springs (49). This apparent anaerobic nitrogenase activity was only observed in a narrow range of the ambient redox potential, based on the occurrence of methanogenesis, and was inhibited by molybdate. The activity was diminished by dispersing the microbial mats with a homogenizer, but was partly recovered by the addition of H_2_, carbon dioxide, and sulfate, which suggested the involvement of chemolithoautotrophic sulfate-reducing bacteria in nitrogen-fixing activities.

As shown in Fig. 3, no NifH sequence closely related to known chemolithoautotrophic sulfate-reducing bacteria was found in the present study. However, NifH-OTUs in group D affiliated with *Nitrospirae* may be candidates for the previously observed activity. The closest relative of NifH-OTUs D1 to D4 was NifH from a sulfate-reducing bacterium in the genus *Thermodesulfovibrio* (86.0 to 96.5% amino acid sequence identity) (Fig. 3 and Table S6). In the genus *Thermodesulfovibrio*, growth was observed up to 70°C in isolates (25, 62). No diazotrophic or autotrophic growth was reported in the genus *Thermodesulfovibrio*, whereas genes related to nitrogen and carbon fixation metabolism (the reductive acetyl-CoA pathway) have been detected in *T. yellowstonii* and its possible nitrogen and carbon fixation abilities have been discussed for this genus (70). NifH-OTU D4 was detected in all samples, and NifH-OTU D1 in particular was highly abundant in white streamers; 9.96% relative abundance in the amplicons of the MehtaF/MehtaR primer set (Fig. 3 and Table S6). A 16S rRNA gene analysis of white streamers identified two reads in the phylum *Nitrospirae*; however, their sequences were only distantly related to known bacteria; the closest isolated relative was *Nitrospira japonica* J1 (NR_114396) with 87.6% sequence identity. NifH-OTUs in group D in the NifH analysis may originate from uncultured species in the phylum *Nitrospirae*, and alternatively, may originate in an as yet phylogenetically undetermined organism.

NifH genes have been reported in chemolithoautotrophic thermophilic sulfate-reducing bacteria in the phylum *Thermodesulfobacteria*; *Thermodesulfatator indicus* DSM 15286 (WP 013907662), *T. autotrophicus* S606 (WP 068540963), and *Thermosulfurimonas dismutans* S95 (WP 068669439). However, despite the detection of 16S rRNA genes for this phylum in microbial communities, affiliated *nifH* sequences were not detected in the present study (Fig. 3).

### Possible aerobic chemolithotrophic nitrogen fixers

In our previous study (49), nitrogenase activity under anaerobic conditions was largely inhibited by molybdate, an inhibitor of sulfate-reducing bacteria. However, low residual nitrogenase activity was still detected after the addition of molybdate, indicating the presence of non-sulfate-reducing, nitrogen-fixing bacteria in the communities. Possible community members that may have been responsible for nitrogenase activity, but were not expected to be sensitive to molybdate inhibition, include members of *Aquificae* and *Caldicellurosiruptor* (*Firmicutes*) (described below in the section of “*Possible fermentative and other nitrogen fixers*”).

An NifH phylogenetic analysis showed NifH-OTUs in group A (*Aquificae*) with a relative abundance between 3.86 and 91.1% in the amplicons of the MehtaF/MehtaR primer set, with an amino acid sequence identity of between 85.1 and 97.4% to those from *Thermocrinis albus* and *Hydrogenobacter thermophilus* in the phylum *Aquificae* (Fig. 4 and Table S6). In group A, NifH-OTU A21 was the most dominant *nifH* sequence in all samples, except for the white streamers (Fig. 3 and Table S6). In the white streamers, NifH-OTU A7 was more abundant, indicating that different diazotrophic *Aquificae* species inhabit different ecological niches in the communities at Nakabusa. Related *nifH* sequences in group A (*Aquificae*) were also reported in analyses of hot spring sediments in Yellowstone National Park (19, 21, 39) and long-term cultivation samples of microbial mats in bioreactors at 65 and 70°C (31) (Fig. 3).

NifH-OTUs A13 to A24 formed a separate cluster within the NifH group A in Fig. 3. In these 13 NifH-OTUs, marked differences at the end 15 amino acids from others were observed due to a frameshift caused by a nucleotide deletion at position 406 (numbering for the *nifH* gene in *A. vinelandii*). The frameshift mutation resulted in a stop codon in A13 to A24 at amino acid positions 139 and/or 149 (numbering for NifH in *A. vinelandii*). Three NifH-OTUs, A13, A14, and A16, have an additional stop codon due to a point mutation at amino acid positions 68, 87, and 115, respectively. These results suggest that NifH-OTUs A13 to A24, including the most dominant NifH-OTU A21, are pseudogenes with no or unknown functions.

*nifH* and 16S rRNA gene analyses showed that bacteria belonging to the phylum *Aquificae* were dominant in all chemosynthetic communities tested in the present study, which is consistent with previous findings (Fig. 2) (11, 48, 67). A metagenomic study on the *Sulfurihydrogenibium*-dominated community of white streamers at Nakabusa suggested that the dominant *Sulfurihydrogenibium* in the phylum *Aquificae* lacked nitrogenase-related genes (67). The order *Aquificales*, which contains *Hydrogenobacter* and *Thermocrinis*, comprises hyperthermophilic sulfur/H_2_-oxidizing aerobic bacteria (29). Two *Aquificales* isolates, *H. thermophilus* TK-6 (WP_012963773) and *T. albus* JCM 11386 (WP_012991466), have been reported to harbor *nifH* genes, but both lack the *nifE* gene coding for a protein involved in the synthesis of the nitrogenase co-factor (2, 59), and their nitrogen-fixing abilities have not yet been demonstrated. Bacterial species related to *Hydrogenobacter* and *Thermocrinis* may fix nitrogen under aerobic conditions using sulfur/H_2_ and oxygen as an electron donor and acceptor, respectively. *In situ*, sulfur is available from the abiotic and/or biotic oxidation of sulfide in the hot spring water without or with the involvement of sulfide-oxidizing bacteria (*e.g. Sulfurihydrogenibium*), and H_2_ is produced by fermentative bacteria in the communities (see below). Furthermore, some members of *Aquificae* have the ability to grow anaerobically using H_2_ as an electron donor and S∘ as an electron acceptor (29). Thus, some of the anaerobic nitrogenase activity of the communities may be achieved by *Aquificae* members. The isolation of these strains, genome sequencing, and physiological analyses are needed to verify whether these organisms harbor the ability to fix nitrogen.

### Possible fermentative and other nitrogen fixers

The NifH sequence of another abundant NifH-OTU in group E (*Firmicutes*) (7.42 to 94.6% of relative abundance in the amplicons of the MehtaF/MehtaR primer set) showed the same sequences as those of *Caldicellulosiruptor hydrothermalis* and *C. kronotskyensis* (Fig. 3 and 4, and Table S6). 16S rRNA gene sequences related to these two species were also found in all four samples (Table S5). Although relative abundance markedly differed between the *nifH* and 16S rRNA genes, sequences that were 100% identical to *Caldicellulosiruptor* sequences were detected in *nifH* and 16S rRNA gene analyses. Comparisons of proportions among samples showed that pale-tan mats contained more abundant NifH-OTU of *Caldicellulosiruptor*; the highest relative abundance of NifH-OTU E3 representing the *Firmicutes* member *Caldicellulosiruptor* sp. was detected in pale-tan mats (94.5% of relative abundance) and the highest relative abundance of the 16S rRNA sequences of *Caldicellulosiruptor* sp. was detected in pale-tan mats (0.55% of relative abundance) (Fig. 3 and 4, and Table S6). *Caldicellulosiruptor* has been isolated from thermophilic microbial communities in various hot springs (5, 22, 43, 46). Nine out of 12 described species with sequenced genomes in the genus *Caldicellulosiruptor* have been reported to possess the *nifH* gene (CP002330, CP002326, CP002216, CP002219, CP002164, CP003001, CP000679, NZ_LACO01000002, and NZ_LACN01000012), whereas nitrogenase activity has not yet been demonstrated in any of these species.

NifH sequences other than those described above (representing chemolithotrophic and fermentative bacteria) and detected in at least two samples were affiliated with *Cyanobacteria*, *Proteobacteria*, and *Chloroflexi* in groups B, C, and G, respectively (Fig. 3 and Table S6). All of these are known members of microbial mat communities found at lower temperatures, but are not expected to exhibit biological activity under the conditions and temperatures sampled in the present study (*i.e.* sulfidic geothermal environments ≥70°C) (12, 16, 23, 38, 58, 66, 73, 74). None of these phyla have been shown to have the capacity to grow under the conditions sampled, and a 16S rRNA amplicon analysis of all samples support low abundance with low read numbers for *Gammaproteobacteria* and the lack of detection of *Cyanobacteria* and *Chloroflexi* (49). Moreover, it remains unclear whether *nifH* in the *Chloroflexi* member *Roseiflexus* (the closest relative of NifH-OTU G1) encodes for a functional nitrogenase component. Nitrogenase activity has not been reported for any *Roseiflexus* spp. culture, and *R. castenholzii* did not grow with dinitrogen as a nitrogen source (70). Although this species contains *nifHDK* genes, it lacks *nifE* and *nifN*. However, this does not necessarily argue against the capability of a functional nitrogenase because a recent study showed nitrogenase activity in an organism lacking *nifEN* in the phylum *Elusimicrobia* (79).

### Microenvironmental diversity and nitrogen fixation

This study found diverse *nifH* sequences in various chemosynthetic microbial communities at 72–77°C in Nakabusa. The phylogenetic diversities of the sequences obtained suggest a diversity of nitrogen fixers on ecological species level within these communities. This may be related to the limited supply of nitrogen compounds as well as the heterogeneity of microenvironments in these communities due to differences in oxygen and sulfide concentrations, as well as other variables. Air-saturated hot water contains approximately 174 μmol L^−1^ of O_2_ at 70°C under the conditions of 21% O_2_ in the atmosphere, which is approximately 60% of that at 20°C (72). Oxygen may be consumed rapidly by aerobic and microaerobic microbes in the highly packed hyperthermophilic communities. Sulfide is consumed with O_2_ and produced within communities (36), leading to changes in the ambient redox potential in the communities. H_2_ is also reported to be produced and consumed in the anoxygenic photosynthetic bacteria-dominated communities in Nakabusa (54), and may play a similar role in electron transfer in the hyperthermophilic chemosynthetic communities analyzed in the present study. The distribution of O_2_, sulfide, and H_2_ within microbial communities may be an important factor for the occurrence of diverse types of nitrogen-fixing bacteria; future studies that link the microanalytical determination of these chemical species to microbial species abundance and activity may contribute to our understanding of how the chemical environment influences microbial communities.

The presence of diverged *nifH* gene sequences does not imply that all or most of the encoded nitrogenases are active *in situ*, nor does the phylogenetic affiliation of these genes definitively correspond to presence in the affiliated organism genomes. Horizontal gene transfer occurs in functional genes, and future genome analyses are required for the placement of *nifH* genes into their genomes. Nitrogenase activity is highly sensitive to O_2_. We previously showed that even under anaerobic conditions, a certain ambient redox potential appeared to be necessary to observe nitrogenase activity in chemosynthetic microbial communities (49). Therefore, not only the biosynthesis of active nitrogen fixing enzymes, but also appropriate metabolic and redox conditions appear to be necessary for active nitrogen fixation. However, a necessary set of genes for nitrogen fixation and the formation of enzymes are prerequisites for nitrogen fixation, and the present results showed that at least two taxa in addition to sulfate reduction-associated nitrogen fixation observed in a previous study (49) may function as nitrogen fixers in the community: aerobic sulfur/hydrogen-oxidizing bacteria in the phylum *Aquificae* and fermentative bacteria in the phylum *Firmicutes*.

## Supplementary Material
