# Supplementary material for: Phylogenetic Diversity of Nitrogenase Reductase Genes and Possible Nitrogen-Fixing Bacteria in Thermophilic Chemosynthetic Microbial Communities in Nakabusa Hot Springs
Source: Microbes Environ. 2018 Nov 7;33(4):357–65. doi: 10.1264/jsme2.ME18030 (PMC6307998; doi:10.1264/jsme2.ME18030)
Supplement: Supplementary file 1 [file 33_357_s1.pdf]

**Table S1.** Coverages of both PolF/PolR and MehtaF/MehtaR primer sets for the representative phylotypes in the *nifH* database.

| Primer sets | Mismatches number[a] | Primers' coverages[b] Cluster I n=242[c] | Cluster II n=57[c] | Cluster III n=144[c] | Cluster IV n=306[c] |
|---|---|---|---|---|---|
| PolF/PolR | 0 | 18.2% | 3.51% | 13.2% | 0.00% |
| | 1 | 59.5% | 26.3% | 30.6% | 0.33% |
| | 2 | 89.3% | 40.4% | 43.1% | 6.54% |
| MehtaF/MehtaR | 0 | 78.9% | 71.9% | 72.2% | 15.4% |
| | 1 | 96.3% | 96.5% | 97.2% | 44.8% |
| | 2 | 99.2% | 96.5% | 99.3% | 71.2% |

**a**, Mismatch number required for covering the sequences are shown; "0", "1", and "2" is the number of mismaches allowed in each primer.
**b**, Primers' coverages were calculated for the 749 representative *nifH* phylotypes listed in Table S3 using the primersearch tool in EMBOSS (Rice *et al*, http://bioinfo.nhri.org.tw/cgi-bin/emboss/primersearch).
**c**, The total number of the representative phylotypes in each cluster used for the calculation. Clusters defined by Zehr *et al*. were used.

Rice, P., L. Longden, and A. Bleasby. 2000. The European Molecular Biology Open Software Suite EMBOSS: The European Molecular Biology Open Software Suite. Trends Genet. 16:276–277.
Zehr, J.P., B.D. Jenkins, S.M. Short, and G.F. Steward. 2003. Nitrogenase gene diversity and microbial community structure: a cross-system comparison. Environ. Microbiol. 5:539–554.

**Table S2**. The number of unique sequences covered by PolF/PolR primer set in the representative phylotypes.

| Mismatches[a] | | Primers' coverages[b] | | | |
|---|---|---|---|---|---|
| | | Cluster I | Cluster II | Cluster III | Cluster IV |
| MehtaF/MehtaR | PolF/PolR | n=242[c] | n=57[c] | n=144[c] | n=306[c] |
| 0 | 0 | 3.72% | 0.00% | 2.08% | 0.0% |
| | 1 | 12.0% | 5.26% | 5.56% | 0.0% |
| | 2 | 17.8% | 7.02% | 9.03% | 2.3% |
| 1 | 0 | 0.41% | 0.00% | 0.00% | 0.0% |
| | 1 | 2.89% | 1.75% | 0.69% | 0.0% |
| | 2 | 3.72% | 1.75% | 1.39% | 1.6% |
| 2 | 0 | 0.00% | 0.00% | 0.00% | 0.0% |
| | 1 | 0.83% | 1.75% | 0.00% | 0.0% |
| | 2 | 0.83% | 1.75% | 0.00% | 0.3% |

**a**, Mismatch number required for covering the sequences were shown; "0", "1", and "2" was the number of mismaches allowed in each primer.

**b**, Primers' coverages were calculated for the 749 representative *nifH* phylotypes listed in Table S3 using the primersearch tool in EMBOSS (Rice *et al*, http://bioinfo.nhri.org.tw/cgi-bin/emboss/primersearch).

**c**, The total number of the representative phylotypes in each cluster used for the calculation. Clusters defined by Zehr *et al*. were used.

Rice, P., L. Longden, and A. Bleasby. 2000. The European Molecular Biology Open Software Suite EMBOSS: The European Molecular Biology Open Software Suite. Trends Genet. 16:276–277.

Zehr, J.P., B.D. Jenkins, S.M. Short, and G.F. Steward. 2003. Nitrogenase gene diversity and microbial community structure: a cross-system comparison. Environ. Microbiol. 5:539–554.

**Table S3**. Sequences used for primers' coverage calculations in Table S1 and S2. The results of coverage for *nifH* primer sets, PolF/PolR and MehtaF/MehtaR, were shown. “Pol” for PolF/PolR and “Mehta” for MehtaF/MehtaR. 749 different phylotypes (<100% matches) in the *nifH* database of Heller *et al*. were selected based on the phylogenetic tree built in ARB (Ludwig *et al*.). Primer coverage calculations were performed using the primersearch tool in EMBOSS (Rice *et al*, http://bioinfo.nhri.org.tw/cgi-bin/emboss/primersearch).

| Clusters | Name[a] | Strain names | Accession num. of NiH | Mismatch num. for cover[b] | |
|---|---|---|---|---|---|
| | | | | Mehta | Pol |
| Cluster I | CntSpec5 | Cyanothece sp. CCY0110 | EAZ92897.1 | 0 | 3 |
| Cluster I | NosSpec9 | Nostoc sp. PCC 7120 | AAA22008.1 | 0 | 1 |
| Cluster I | CntSpec7 | Cyanothece sp. ATCC 51142 | ACB49910.1 | 0 | 3 |
| Cluster I | CntSpe10 | Cyanothece sp. ATCC 51142 | AAW56988.1 | 0 | 3 |
| Cluster I | CnbEndos | cyanobacterium endosymbiont of Rhopalodia gibba | AAW57049.1 | 0 | 3 |
| Cluster I | FranAlni | Frankia alni ACN14a | CAJ65436.1 | 0 | 0 |
| Cluster I | LynSpec3 | Lyngbya sp. PCC 8106 | EAW37219.1 | 0 | 2 |
| Cluster I | NodSpum7 | Nodularia spumigena CCY9414 | EAW46334.1 | 0 | 1 |
| Cluster I | FraSpe48 | Frankia sp. EAN1pec | ABW16215.1 | 0 | 0 |
| Cluster I | NosSpe13 | Nostoc sp. PCC 7120 (Anabaena sp. PCC 7120) | BAB73411.1 | 0 | 1 |
| Cluster I | NosSpe14 | Nostoc sp. PCC 7120 | NP_484917.1 | 0 | 2 |
| Cluster I | NosPunc2 | Nostoc punctiforme PCC 73102 (Nostoc punctiforme ATCC 29133) | ACC79194.1 | 0 | 1 |
| Cluster I | NosPunc3 | Nostoc punctiforme PCC 73102 (Nostoc punctiforme ATCC 29133) | ACC79763.1 | 0 | 1 |
| Cluster I | NosPunc4 | Nostoc punctiforme PCC 73102 (Nostoc punctiforme ATCC 29133) | ACC84196.1 | 0 | 1 |
| Cluster I | SncSpeci | Synechococcus sp. PCC 7335 | EDX87850.1 | 0 | 1 |
| Cluster I | CntSpe11 | Cyanothece sp. PCC 7822 | EDX97588.1 | 0 | 2 |
| Cluster I | CntSpe16 | Cyanothece sp. PCC 7424 | ACK70547.1 | 0 | 3 |
| Cluster I | CntSpe19 | Cyanothece sp. PCC 7425 | ACL44722.1 | 1 | 2 |
| Cluster I | UncNos27 | 'Nostoc azollae' 0708 | EEG00294.1 | 0 | 2 |
| Cluster I | UncNos28 | 'Nostoc azollae' 0708 | EEG00728.1 | 0 | 1 |
| Cluster I | CroWats3 | Crocosphaera watsonii WH 8501 (Synechocystis sp. WH 8501) | EAM50501.1 | 0 | 1 |
| Cluster I | AnaVari6 | Anabaena variabilis ATCC 29413 (Anabaena flos-aquae UTEX 1444) | ABA23521.1 | 0 | 1 |
| Cluster I | AnaVari7 | Anabaena variabilis ATCC 29413 (Anabaena flos-aquae UTEX 1444) | ABA23658.1 | 0 | 2 |
| Cluster I | AnaVari8 | Anabaena variabilis ATCC 29413 (Anabaena flos-aquae UTEX 1444) | ABA23846.1 | 0 | 1 |
| Cluster I | AnaVari9 | Anabaena variabilis ATCC 29413 (Anabaena flos-aquae UTEX 1444) | ABA24076.1 | 0 | 1 |
| Cluster I | FraSpe53 | Frankia sp. CcI3 | ABD13834.1 | 0 | 0 |
| Cluster I | SncSpec6 | Synechococcus sp. JA-3-3Ab | YP_475238.1 | 0 | 1 |
| Cluster I | TriEryth | Trichodesmium erythraeum IMS101 | ABG53144.1 | 0 | 2 |
| Cluster I | RhdPal18 | Rhodopseudomonas palustris BisA53 | ABJ08453.1 | 0 | 1 |
| Cluster I | MgnSpeci | Magnetococcus sp. MC-1 | ABK43713.1 | 0 | 1 |
| Cluster I | PlbPropi | Pelobacter propionicus DSM 2379 | ABL01060.1 | 0 | 1 |
| Cluster I | AzrSpec4 | Azoarcus sp. BH72 | YP_932042.1 | 0 | 1 |
| Cluster I | HlhHalop | Halorhodospira halophila SL1 | ABM61068.1 | 0 | 0 |
| Cluster I | PolNaph2 | Polaromonas naphthalenivorans CJ2 | YP_982573.1 | 0 | 0 |
| Cluster I | BurViet6 | Burkholderia vietnamiensis G4 | YP_001115195.1 | 0 | 1 |
| Cluster I | MesoLot6 | Mesorhizobium loti MAFF303099 | BAB52275.1 | 0 | 1 |
| Cluster I | RhbSpha6 | Rhodobacter sphaeroides ATCC 17025 | ABP70147.1 | 1 | 2 |
| Cluster I | PseStut2 | Pseudomonas stutzeri A1501 | ABP79021.1 | 1 | 0 |
| Cluster I | BraSpe41 | Bradyrhizobium sp. ORS278 | CAL79117.1 | 0 | 1 |
| Cluster I | GeoUran3 | Geobacter uraniireducens Rf4 | ABQ25379.1 | 0 | 0 |
| Cluster I | SinMedi5 | Sinorhizobium medicae WSM419 | YP_001314762.1 | 1 | 1 |
| Cluster I | AnmSpeci | Anaeromyxobacter sp. Fw109-5 | ABS27227.1 | 1 | 0 |
| Cluster I | XanAuto4 | Xanthobacter autotrophicus Py2 | YP_001415004.1 | 1 | 0 |
| Cluster I | AzhCaul5 | Azorhizobium caulinodans ORS 571 | BAF87039.1 | 1 | 0 |
| Cluster I | AzhCaul6 | Azorhizobium caulinodans ORS 571 | BAF89441.1 | 1 | 0 |
| Cluster I | HelMode3 | Heliobacterium modesticaldum Ice1 | ABZ83695.1 | 0 | 0 |
| Cluster I | MlbSpec2 | Methylobacterium sp. 4-46 | YP_001770351.1 | 3 | 1 |
| Cluster I | CupTaiw2 | Cupriavidus taiwanensis | CAP64045.1 | 0 | 2 |
| Cluster I | LphChol2 | Leptothrix cholodnii SP-6 | YP_001790463.1 | 0 | 1 |
| Cluster I | BeiIndi2 | Beijerinckia indica subsp. indica ATCC 9039 | ACB94126.1 | 0 | 1 |
| Cluster I | BurPhym3 | Burkholderia phymatum STM815 | ACC76639.1 | 0 | 2 |
| Cluster I | BurPhym4 | Burkholderia phymatum STM815 | ACC76684.1 | 0 | 2 |
| Cluster I | MlcInfe2 | Methylacidiphilum infernorum V4 | YP_001940528.1 | 0 | 2 |
| Cluster I | GeoLovle | Geobacter lovleyi SZ | ACD94376.1 | 0 | 1 |
| Cluster I | GeoLovl2 | Geobacter lovleyi SZ | ACD94940.1 | 0 | 2 |
| Cluster I | RhdPal24 | Rhodopseudomonas palustris TIE-1 | YP_001994065.1 | 0 | 1 |
| Cluster I | AnmSpec3 | Anaeromyxobacter sp. K | ACG74424.1 | 1 | 1 |
| Cluster I | GeoBemid | Geobacter bemidjiensis Bem | ACH39087.1 | 0 | 1 |
| Cluster I | KlePneu4 | Klebsiella pneumoniae 342 | YP_002237565.1 | 0 | 0 |
| Cluster I | ThrYello | Thermodesulfovibrio yellowstonii DSM 11347 | ACI21914.1 | 1 | 3 |

| | | | | | |
|---|---|---|---|---|---|
| Cluster I | GluDiaz5 | Gluconacetobacter diazotrophicus PAl 5 | ACI51349.1 | 0 | 2 |
| Cluster I | RhiLeg14 | Rhizobium leguminosarum bv. trifolii WSM2304 | YP_002279010.1 | 1 | 2 |
| Cluster I | RhsCente | Rhodospirillum centenum SW (Rhodocista centenaria SW) | ACJ01032.1 | 0 | 2 |
| Cluster I | RhizEt53 | Rhizobium etli CFN 42 | NP_659836.1 | 2 | 1 |
| Cluster I | MtcSilv5 | Methylocella silvestris BL2 | ACK52517.1 | 0 | 1 |
| Cluster I | AciFerr4 | Acidithiobacillus ferrooxidans ATCC 23270 | ACK79918.1 | 1 | 1 |
| Cluster I | DstHaf12 | Desulfitobacterium hafniense DCB-2 | ACL19109.1 | 0 | 1 |
| Cluster I | MlbNodul | Methylobacterium nodulans ORS 2060 | ACL58883.1 | 3 | 1 |
| Cluster I | GeoSpec3 | Geobacter sp. FRC-32 | ACM20925.1 | 0 | 0 |
| Cluster I | RhbSph11 | Rhodobacter sphaeroides KD131 | YP_002526237.1 | 1 | 2 |
| Cluster I | AztVine8 | Azotobacter vinelandii DJ | ACO76403.1 | 0 | 1 |
| Cluster I | AztVine9 | Azotobacter vinelandii DJ | ACO76527.1 | 0 | 1 |
| Cluster I | DenAcet2 | Denitrovibrio acetiphilus DSM 12809 | ZP_03905880.1 | 0 | 2 |
| Cluster I | TlmAuens | Tolumonas auensis DSM 9187 | ACQ92279.1 | 0 | 2 |
| Cluster I | TerTurne | Teredinibacter turnerae T7901 | ACR12580.1 | 0 | 2 |
| Cluster I | RhiLeg15 | Rhizobium leguminosarum bv. trifolii WSM1325 | ACS59148.1 | 0 | 2 |
| Cluster I | ZymMobil | Zymomonas mobilis subsp. mobilis ATCC 10988 | EER62613.1 | 0 | 3 |
| Cluster I | AllVinos | Allochromatium vinosum DSM 180 | EER67145.1 | 0 | 1 |
| Cluster I | DicDadan | Dickeya dadantii Ech703 | ACS84302.1 | 2 | 1 |
| Cluster I | LepFerr2 | Leptospirillum ferrodiazotrophum (mine drainage metagenome) | EES53484.1 | 1 | 1 |
| Cluster I | GeoSpec5 | Geobacter sp. M21 | ACT18197.1 | 0 | 0 |
| Cluster I | PanSpec2 | Pantoea sp. At-9b | EEW04348.1 | 0 | 1 |
| Cluster I | BraJap17 | Bradyrhizobium japonicum USDA 110 | NP_768409.1 | 1 | 2 |
| Cluster I | RhdPal26 | Rhodopseudomonas palustris DX-1 | EFC25536.1 | 0 | 1 |
| Cluster I | SinMel28 | Sinorhizobium meliloti AK83 | EFN22758.1 | 0 | 1 |
| Cluster I | RhdPal32 | Rhodopseudomonas palustris CGA009 | NP_946726.1 | 0 | 2 |
| Cluster I | GeoSulfu | Geobacter sulfurreducens PCA | AAR36215.1 | 0 | 1 |
| Cluster I | PecAtros | Pectobacterium atrosepticum SCRI1043 | CAG75856.1 | 2 | 2 |
| Cluster I | MhlCaps2 | Methylococcus capsulatus str. Bath | AAU90633.1 | 0 | 0 |
| Cluster I | ZymMobi4 | Zymomonas mobilis subsp. mobilis ZM4 | YP_163558.1 | 0 | 3 |
| Cluster I | DecAroma | Dechloromonas aromatica RCB | AAZ46164.1 | 0 | 0 |
| Cluster I | PlbCarbi | Pelobacter carbinolicus DSM 2380 | ABA89338.1 | 0 | 0 |
| Cluster I | MagMagn3 | Magnetospirillum magneticum AMB-1 | BAE50378.1 | 0 | 1 |
| Cluster I | RhdPal37 | Rhodopseudomonas palustris BisB18 | ABD89986.1 | 0 | 1 |
| Cluster I | RhdPal41 | Rhodopseudomonas palustris BisB5 | ABE38311.1 | 0 | 1 |
| Cluster I | BurXeno2 | Burkholderia xenovorans LB400 | YP_553849.1 | 0 | 0 |
| Cluster I | DlfAceto | Desulfuromonas acetoxidans DSM 684 | EAT15955.1 | 0 | 1 |
| Cluster I | WolSucci | Wolinella succinogenes | CAE10460.1 | 0 | 2 |
| Cluster I | BurFerra | Burkholderia ferrariae | ABO64209.1 | 1 | 1 |
| Cluster I | BurTrop3 | Burkholderia tropica | ABO64210.1 | 1 | 1 |
| Cluster I | BurUnama | Burkholderia unamae | ABO64213.1 | 1 | 2 |
| Cluster I | BurXeno3 | Burkholderia xenovorans | ABO64215.1 | 1 | 0 |
| Cluster I | BurSilva | Burkholderia silvatlantica | ABO64217.1 | 0 | 1 |
| Cluster I | BurSilv2 | Burkholderia silvatlantica | ABO64218.1 | 1 | 1 |
| Cluster I | BurViet7 | Burkholderia vietnamiensis | ABO64219.1 | 0 | 1 |
| Cluster I | BurViet8 | Burkholderia vietnamiensis | ABO64221.1 | 1 | 1 |
| Cluster I | BurUnam2 | Burkholderia unamae | ABO64212.1 | 0 | 2 |
| Cluster I | BurUnam3 | Burkholderia unamae MTI-641 | ABO64214.1 | 0 | 2 |
| Cluster I | BurCaryo | Burkholderia caryophylli | ABO64216.1 | 0 | 1 |
| Cluster I | AciFerri | Acidithiobacillus ferrivorans SS3 | EGK89721.1 | 0 | 0 |
| Cluster I | CntSpe21 | Cyanothece sp. PCC 8802 | ACV00712.1 | 0 | 2 |
| Cluster I | CanAccum | Candidatus Accumulibacter phosphatis clade IIA str. UW-1 | ACV37675.1 | 0 | 1 |
| Cluster I | ZymMobi5 | Zymomonas mobilis subsp. mobilis NCIMB 11163 | ACV75951.1 | 0 | 3 |
| Cluster I | RhbSpec2 | Rhodobacter sp. SW2 | EEW25375.1 | 0 | 2 |
| Cluster I | CylRac72 | Cylindrospermopsis raciborskii CS-505 | EFA68738.1 | 0 | 1 |
| Cluster I | CnbUCYNA | cyanobacterium UCYN-A | YP_003421696.1 | 0 | 3 |
| Cluster I | FraSpe77 | Frankia sp. EUN1f | EFC83885.1 | 0 | 0 |
| Cluster I | HdrTher2 | Hydrogenobacter thermophilus TK-6 | YP_003432794.1 | 0 | 2 |
| Cluster I | AzoSpe28 | Azospirillum sp. B510 | BAI71409.1 | 0 | 1 |
| Cluster I | KleVari6 | Klebsiella variicola At-22 | YP_003438540.1 | 0 | 0 |
| Cluster I | ThmAlbus | Thermocrinis albus DSM 14484 | ADC89059.1 | 0 | 2 |
| Cluster I | SidLitho | Sideroxydans lithotrophicus ES-1 | ADE11119.1 | 1 | 0 |
| Cluster I | RhbCaps5 | Rhodobacter capsulatus SB 1003 | ADE84337.1 | 0 | 2 |
| Cluster I | BurSp150 | Burkholderia sp. CCGE1002 | ADG20727.1 | 0 | 2 |
| Cluster I | BurSp152 | Burkholderia sp. Ch1-1 | EFG73464.1 | 0 | 0 |
| Cluster I | ArcNitr4 | Arcobacter nitrofigilis DSM 7299 | ADG91719.1 | 1 | 3 |

| | | | | | |
|---|---|---|---|---|---|
| Cluster I | MtsTric6 | Methylosinus trichosporium OB3b | EFH04962.1 | 0 | 0 |
| Cluster I | UncNos37 | 'Nostoc azollae' 0708 | ADI65368.1 | 0 | 2 |
| Cluster I | HerSero6 | Herbaspirillum seropedicae SmR1 | ADJ64345.1 | 0 | 0 |
| Cluster I | OscSpec4 | Oscillatoria sp. PCC 6506 (Oscillatoria sp. PCC 9029) | CBN57732.1 | 0 | 3 |
| Cluster I | DicDada6 | Dickeya dadantii 3937 | YP_003884784.1 | 0 | 1 |
| Cluster I | RhmVanni | Rhodomicrobium vannielii ATCC 17100 | ADP71189.1 | 0 | 1 |
| Cluster I | CltNitr2 | Calditerrivibrio nitroreducens DSM 19672 | YP_004050758.1 | 1 | 3 |
| Cluster I | SlrKuji2 | Sulfuricurvum kujiense DSM 16994 | YP_004060362.1 | 1 | 2 |
| Cluster I | DspIndi2 | Desulfurispirillum indicum S5 | YP_004111991.1 | 0 | 2 |
| Cluster I | MesCic17 | Mesorhizobium ciceri biovar biserrulae WSM1271 | YP_004144892.1 | 0 | 2 |
| Cluster I | GeoSpe12 | Geobacter sp. M18 | ADW13325.1 | 0 | 0 |
| Cluster I | MtlSpec7 | Methylocystis sp. ATCC 49242 | ZP_08073552.1 | 0 | 0 |
| Cluster I | SnbGlyc2 | Syntrophobotulus glycolicus DSM 8271 | ADY57111.1 | 1 | 2 |
| Cluster I | NovNitr3 | Novosphingobium nitrogenifigens DSM 19370 | ZP_08207527.1 | 0 | 1 |
| Cluster I | RubBenz2 | Rubrivivax benzoatilyticus JA2 | ZP_08402608.1 | 0 | 0 |
| Cluster I | MtmMeth4 | Methylomonas methanica MC09 | YP_004512383.1 | 0 | 2 |
| Cluster I | FraSymb4 | Frankia symbiont of Datisca glomerata | YP_004584062.1 | 0 | 1 |
| Cluster I | ZymMobi9 | Zymomonas mobilis subsp. pomaceae ATCC 29192 | AEI38202.1 | 1 | 2 |
| Cluster I | MesOppo5 | Mesorhizobium opportunistum WSM2075 | YP_004614826.1 | 0 | 2 |
| Cluster I | HphSpec2 | Hyphomicrobium sp. MC1 | CCB66897.1 | 0 | 2 |
| Cluster I | HphSpec3 | Hyphomicrobium sp. MC1 | YP_004677413.1 | 0 | 2 |
| Cluster I | TrhDrew2 | Thiorhodococcus drewsii AZ1 | EGV30156.1 | 0 | 1 |
| Cluster I | MtbTund7 | Methylobacter tundripaludum SV96 | EGW20197.1 | 0 | 1 |
| Cluster I | DslSpec2 | Desulfosporosinus sp. OT | EGW39330.1 | 0 | 3 |
| Cluster I | ThcMari2 | Thiocapsa marina 5811 | ZP_08769524.1 | 0 | 1 |
| Cluster I | MrcPurp4 | Marichromatium purpuratum 984 | ZP_08776218.1 | 0 | 1 |
| Cluster I | ArcSpeci | Arcobacter sp. L | BAK71909.1 | 1 | 3 |
| Cluster I | AzoAmaz3 | Azospirillum amazonense Y2 | ZP_08870627.1 | 0 | 1 |
| Cluster I | ThhSibi3 | Thiorhodospira sibirica ATCC 700588 | ZP_08921284.1 | 0 | 1 |
| Cluster I | ThtViol2 | Thiocystis violascens DSM 198 | ZP_08926951.1 | 0 | 0 |
| Cluster I | ThvSpec2 | Thiorhodovibrio sp. 970 | ZP_08941328.1 | 0 | 0 |
| Cluster I | DslMeri2 | Desulfosporosinus meridiei DSM 13257 | ZP_08980127.1 | 0 | 2 |
| Cluster I | FisSpec4 | Fischerella sp. JSC-11 | ZP_08987693.1 | 0 | 1 |
| Cluster I | MesAust2 | Mesorhizobium australicum WSM2073 | ZP_08993929.1 | 0 | 2 |
| Cluster I | BreSpec2 | Brenneria sp. EniD312 | ZP_09015409.1 | 1 | 1 |
| Cluster I | MesAmo18 | Mesorhizobium amorphae CCNWGS0123 | ZP_09085384.1 | 0 | 2 |
| Cluster I | CroWats5 | Crocosphaera watsonii WH 0003 | EHJ12384.1 | 0 | 1 |
| Cluster I | AzrSpec7 | Azoarcus sp. KH32C | BAL23130.1 | 0 | 1 |
| Cluster I | AzrSpec8 | Azoarcus sp. KH32C | BAL26358.1 | 0 | 1 |
| Cluster I | DchSuil2 | Dechlorosoma suillum PS | AEV25360.1 | 0 | 0 |
| Cluster I | SinFre46 | Sinorhizobium fredii HH103 | CCE98881.1 | 0 | 1 |
| Cluster I | BraSp335 | Bradyrhizobium sp. ORS 285 | CCD84359.1 | 0 | 1 |
| Cluster I | BraSp342 | Bradyrhizobium sp. ORS 285 | ZP_09471847.1 | 0 | 1 |
| Cluster I | GeoMeta3 | Geobacter metallireducens RCH3 | EHP84799.1 | 0 | 0 |
| Cluster I | AzoLipo9 | Azospirillum lipoferum 4B | YP_005038294.1 | 0 | 1 |
| Cluster I | PaeTerr2 | Paenibacillus terrae HPL-003 | YP_005075592.1 | 0 | 2 |
| Cluster I | DslYoun2 | Desulfosporosinus youngiae DSM 17734 | EHQ91738.1 | 1 | 3 |
| Cluster I | BraSp344 | Bradyrhizobium sp. WSM471 | EHR00803.1 | 0 | 0 |
| Cluster I | DslOrie3 | Desulfosporosinus orientis DSM 765 | YP_004972933.1 | 0 | 2 |
| Cluster I | PaeSpe35 | Paenibacillus sp. Aloe-11 | EHS58803.1 | 0 | 2 |
| Cluster I | BrkBact2 | Burkholderiales bacterium JOSHI_001 | ZP_09750647.1 | 0 | 0 |
| Cluster I | KleOxyt3 | Klebsiella oxytoca KCTC 1686 | YP_005020938.1 | 0 | 0 |
| Cluster I | PhaMolis | Phaeospirillum molischianum DSM 120 | CCG41896.1 | 0 | 3 |
| Cluster I | ZymMob12 | Zymomonas mobilis subsp. mobilis ATCC 10988 | YP_005621605.1 | 0 | 3 |
| Cluster I | MlcFuma2 | Methylacidiphilum fumariolicum SolV | ZP_10016521.1 | 0 | 2 |
| Cluster I | RhsRub12 | Rhodospirillum rubrum F11 | YP_006047308.1 | 0 | 2 |
| Cluster I | ThxNive2 | Thiothrix nivea DSM 5205 | ZP_10105314.1 | 0 | 2 |
| Cluster I | BeggAlb2 | Beggiatoa alba B18LD | ZP_10114906.1 | 0 | 1 |
| Cluster I | RahAqua2 | Rahnella aquatilis HX2 | YP_006258984.1 | 2 | 2 |
| Cluster I | McvSpec5 | Microvirga sp. WSM3557 | EIM30737.1 | 1 | 1 |
| Cluster I | SlfBarn2 | Sulfurospirillum barnesii SES-3 | YP_006403911.1 | 0 | 3 |
| Cluster I | FraSp105 | Frankia sp. QA3 | EIV95132.1 | 0 | 0 |
| Cluster I | AzoBra48 | Azospirillum brasilense Sp245 | YP_005030951.1 | 0 | 1 |
| Cluster I | DslAcid2 | Desulfosporosinus acidiphilus SJ4 | YP_006468233.1 | 0 | 3 |
| Cluster I | RhiLe213 | Rhizobium leguminosarum bv. trifolii WU95 | EIW40458.1 | 0 | 2 |
| Cluster I | RhiLe215 | Rhizobium leguminosarum bv. trifolii WSM597 | EJB07190.1 | 1 | 2 |

| | | | | | |
|---|---|---|---|---|---|
| Cluster I | FraSp106 | Frankia sp. QA3 | ZP_10311456.1 | 0 | 0 |
| Cluster I | RhiLe216 | Rhizobium leguminosarum bv. viciae WSM1455 | EJC64806.1 | 0 | 3 |
| Cluster I | RhiLe217 | Rhizobium leguminosarum bv. trifolii WSM2012 | EJC75087.1 | 0 | 2 |
| Cluster I | RhiLe218 | Rhizobium leguminosarum bv. trifolii WSM2297 | EJC80732.1 | 0 | 2 |
| Cluster I | NovSpec2 | Novosphingobium sp. Rr 2-17 | ZP_10360790.1 | 0 | 1 |
| Cluster I | EntRadic | Enterobacter radicincitans DSM 16656 | EJI87739.1 | 0 | 1 |
| Cluster I | MtlSpec8 | Methylocystis sp. SC2 | CCJ08074.1 | 1 | 0 |
| Cluster I | RhiSp148 | Rhizobium sp. CCGE 510 | EJT01245.1 | 2 | 1 |
| Cluster I | RhvSpec2 | Rhodovulum sp. PH10 | EJW09446.1 | 0 | 1 |
| Cluster I | BurPheno | Burkholderia phenoliruptrix BR3459a | AFT90593.1 | 0 | 2 |
| Cluster I | RhiMeso2 | Rhizobium mesoamericanum STM3625 | ZP_11269028.1 | 1 | 1 |
| Cluster I | PseStu21 | Pseudomonas stutzeri KOS6 | EKN58682.1 | 2 | 0 |
| Cluster I | DhlSpec4 | Dehalobacter sp. CF | YP_006914006.1 | 1 | 3 |
| Cluster I | OstCyan3 | Oscillatoriales cyanobacterium JSC-12 | ZP_11393330.1 | 1 | 3 |
| Cluster I | KleOxyt9 | Klebsiella oxytoca 10-5250 | ZP_17112338.1 | 0 | 0 |
| Cluster I | LptSpec9 | Leptolyngbya sp. PCC 7375 | EKV00060.1 | 0 | 2 |
| Cluster I | LptSpe10 | Leptolyngbya sp. PCC 6406 | ELR91185.1 | 0 | 1 |
| Cluster I | SerSpeci | Serratia sp. ATCC 39006 | AGU89250.1 | 0 | 1 |
| Cluster I | CnsSalin | Caenispirillum salinarum AK4 | EKV30731.1 | 2 | 1 |
| Cluster I | CalSpec9 | Calothrix sp. PCC 7507 | AFY30594.1 | 0 | 1 |
| Cluster I | CalSpe10 | Calothrix sp. PCC 7507 | AFY35853.1 | 0 | 0 |
| Cluster I | NosSpe52 | Nostoc sp. PCC 7107 | AFY43944.1 | 0 | 2 |
| Cluster I | NosSpe53 | Nostoc sp. PCC 7524 (Nostoc sp. ATCC 29411) | AFY51257.1 | 1 | 2 |
| Cluster I | RivSpeci | Rivularia sp. PCC 7116 | AFY58697.1 | 0 | 3 |
| Cluster I | NosSpe54 | Nostoc sp. PCC 7107 | YP_007048826.1 | 0 | 1 |
| Cluster I | PlrSpec3 | Pleurocapsa sp. PCC 7327 | YP_007082289.1 | 0 | 2 |
| Cluster I | ChcTher3 | Chroococcidiopsis thermalis PCC 7203 | YP_007093844.1 | 1 | 1 |
| Cluster I | ClnStag2 | Cylindrospermum stagnale PCC 7417 | AFZ22411.1 | 0 | 0 |
| Cluster I | ClnStag3 | Cylindrospermum stagnale PCC 7417 | AFZ22902.1 | 0 | 1 |
| Cluster I | ClnStag4 | Cylindrospermum stagnale PCC 7417 | AFZ27429.1 | 0 | 1 |
| Cluster I | MccSpec3 | Microcoleus sp. PCC 7113 | YP_007120584.1 | 0 | 1 |
| Cluster I | HalSpec3 | Halothece sp. PCC 7418 (Aphanothece halophytica 7418) | YP_007168168.1 | 0 | 2 |
| Cluster I | ThfMobil | Thioflavicoccus mobilis 8321 | AGA91923.1 | 0 | 1 |
| Cluster I | AnaCyli5 | Anabaena cylindrica PCC 7122 | YP_007157762.1 | 0 | 2 |
| Cluster I | XenSpec2 | Xenococcus sp. PCC 7305 | ELS00719.1 | 0 | 2 |
| Cluster I | MagSpec2 | Magnetospirillum sp. SO-1 | EME68108.1 | 0 | 2 |
| Cluster I | CloUltun | Clostridium ultunense Esp | CCQ92501.1 | 0 | 2 |
| Cluster I | BraOlig4 | Bradyrhizobium oligotrophicum S58 | YP_007511903.1 | 0 | 1 |
| Cluster I | MesMetal | Mesorhizobium metallidurans STM 2683 | CCV09310.1 | 0 | 2 |
| Cluster I | MesoLo10 | Mesorhizobium loti NZP2037 | BAN09663.1 | 0 | 1 |
| Cluster I | UncSulf2 | uncultured Sulfuricurvum sp. RIFRC-1 | YP_007528318.1 | 1 | 3 |
| Cluster I | RhiFreir | Rhizobium freirei PRF 81 | ENN84831.1 | 1 | 1 |
| Cluster I | HerFris3 | Herbaspirillum frisingense GSF30 | EOA05112.1 | 0 | 1 |
| Cluster I | AgaAlbus | Agarivorans albus MKT 106 | GAD03531.1 | 0 | 3 |
| Cluster I | EntSpe10 | Enterobacter sp. R4-368 | YP_008106492.1 | 1 | 1 |
| Cluster I | RhiGraha | Rhizobium grahamii CCGE 502 | EPE93655.1 | 1 | 2 |
| Cluster I | VibNatr2 | Vibrio natriegens NBRC 15636 = ATCC 14048 = DSM 759 | EPM41546.1 | 0 | 2 |
| Cluster I | PhaFulv3 | Phaeospirillum fulvum MGU-K5 | EPY01003.1 | 0 | 1 |
| Cluster I | PhaFulv4 | Phaeospirillum fulvum MGU-K5 | EPY03425.1 | 0 | 1 |
| Cluster I | LepSpeci | Leptospirillum sp. Group IV 'UBA BS' (mine drainage metagenome) | EQD24965.1 | 0 | 2 |
| Cluster II | RhdPal17 | Rhodopseudomonas palustris BisA53 | ABJ07810.1 | 0 | 3 |
| Cluster II | MhnMarip | Methanococcus maripaludis C5 | ABO34969.1 | 0 | 3 |
| Cluster II | CloKlu17 | Clostridium kluyveri DSM 555 | EDK33790.1 | 1 | 3 |
| Cluster II | MhnVann4 | Methanococcus vannielii SB | ABR53979.1 | 1 | 3 |
| Cluster II | MhnAeol4 | Methanococcus aeolicus Nankai-3 | ABR57010.1 | 0 | 3 |
| Cluster II | MhnMari3 | Methanococcus maripaludis C7 | ABR65168.1 | 0 | 3 |
| Cluster II | MhbTher3 | (Methanobacterium thermoautotrophicum str. deltaH) | NP_276673.1 | 0 | 3 |
| Cluster II | MhnMar10 | Methanococcus maripaludis C6 | YP_001549846.1 | 0 | 3 |
| Cluster II | CanDesul | Candidatus Desulforudis audaxviator MP104C | ACA58711.1 | 0 | 3 |
| Cluster II | RhdPal21 | Rhodopseudomonas palustris TIE-1 | ACF00152.1 | 0 | 3 |
| Cluster II | ChhThal3 | Chloroherpeton thalassium ATCC 35110 | ACF14285.1 | 0 | 3 |
| Cluster II | MetAcet5 | Methanosarcina acetivorans C2A | AAM04624.1 | 1 | 3 |
| Cluster II | MetAcet6 | Methanosarcina acetivorans C2A | AAM04632.1 | 1 | 3 |
| Cluster II | RhbSpha9 | Rhodobacter sphaeroides KD131 | ACM03752.1 | 0 | 3 |
| Cluster II | OpiBact3 | Opitutaceae bacterium TAV2 | EEG18913.1 | 0 | 3 |
| Cluster II | AztVin10 | Azotobacter vinelandii DJ | ACO81000.1 | 0 | 3 |

| | | | | | |
|---|---|---|---|---|---|
| Cluster II | MtdInfe3 | Methanocaldococcus infernus ME | EER82084.1 | 3 | 3 |
| Cluster II | DicDada2 | Dickeya dadantii Ech703 | ACS86387.1 | 0 | 3 |
| Cluster II | RhdPal25 | Rhodopseudomonas palustris DX-1 | EFC24679.1 | 0 | 3 |
| Cluster II | EthHarb3 | Ethanoligenens harbinense YUAN-3 | EFD40330.1 | 0 | 3 |
| Cluster II | MhmOkin3 | Methanothermococcus okinawensis IH1 | EFL48887.1 | 0 | 3 |
| Cluster II | MhnMar14 | Methanococcus maripaludis S2 | NP_987973.1 | 0 | 3 |
| Cluster II | MetBar11 | Methanosarcina barkeri str. Fusaro | AAZ71206.1 | 0 | 3 |
| Cluster II | RhsRubr5 | Rhodospirillum rubrum ATCC 11170 | ABC22196.1 | 0 | 3 |
| Cluster II | RhdPal38 | Rhodopseudomonas palustris BisB18 | ABD90206.1 | 0 | 3 |
| Cluster II | MtdVulc2 | Methanocaldococcus vulcanius M7 | ACX72939.1 | 1 | 3 |
| Cluster II | MtdSpeci | Methanocaldococcus sp. FS406-22 | ADC68732.1 | 1 | 3 |
| Cluster II | RhbCaps6 | Rhodobacter capsulatus SB 1003 | ADE84350.1 | 0 | 3 |
| Cluster II | SnpLipoc | Syntrophothermus lipocalidus DSM 12680 | ADI02873.1 | 1 | 3 |
| Cluster II | MtnMarbu | Methanothermobacter marburgensis str. Marburg | ADL57755.1 | 0 | 3 |
| Cluster II | RhmVann2 | Rhodomicrobium vannielii ATCC 17100 | ADP72548.1 | 0 | 3 |
| Cluster II | PalPropi | Paludibacter propionicigenes WB4 | ADQ79512.1 | 0 | 3 |
| Cluster II | SnbGlyc6 | Syntrophobotulus glycolicus DSM 8271 | YP_004267124.1 | 0 | 3 |
| Cluster II | MhbSpec2 | Methanobacterium sp. AL-21 | ADZ10041.1 | 1 | 3 |
| Cluster II | MnrIgne3 | Methanotorris igneus Kol 5 | AEF97268.1 | 0 | 3 |
| Cluster II | DesKuzn6 | Desulfotomaculum kuznetsovii DSM 6115 | YP_004518631.1 | 0 | 3 |
| Cluster II | MhbSpe12 | Methanobacterium sp. SWAN-1 | YP_004520824.1 | 0 | 3 |
| Cluster II | ThsIndic | Thermodesulfatator indicus DSM 15286 | AEH44920.1 | 0 | 3 |
| Cluster II | ZymMobi8 | Zymomonas mobilis subsp. pomaceae ATCC 29192 | AEI37864.1 | 0 | 3 |
| Cluster II | DslSpeci | Desulfosporosinus sp. OT | EGW37575.1 | 0 | 3 |
| Cluster II | TrhDrew4 | Thiorhodococcus drewsii AZ1 | ZP_08824429.1 | 0 | 3 |
| Cluster II | CloSpe23 | Clostridium sp. DL-VIII | EHJ00773.1 | 1 | 3 |
| Cluster II | OpiBact7 | Opitutaceae bacterium TAV5 | EHP33172.1 | 0 | 3 |
| Cluster II | MnrForm8 | Methanotorris formicicus Mc-S-70 | ZP_09708037.1 | 0 | 3 |
| Cluster II | TrePri13 | Treponema primitia ZAS-1 | ZP_09716421.1 | 1 | 3 |
| Cluster II | AceWood3 | Acetobacterium woodii DSM 1030 | AFA47495.1 | 0 | 3 |
| Cluster II | DsfSpec8 | Desulfovibrio sp. U5L | EIG54448.1 | 0 | 3 |
| Cluster II | PlsFer13 | Pelosinus fermentans A11 | EIW26096.1 | 0 | 3 |
| Cluster II | EntRadi3 | Enterobacter radicincitans DSM 16656 | ZP_10488873.1 | 1 | 3 |
| Cluster II | RhvSpec3 | Rhodovulum sp. PH10 | EJW10892.1 | 0 | 3 |
| Cluster II | MhbForm2 | Methanobacterium formicicum DSM 3637 | EKF87100.1 | 1 | 3 |
| Cluster II | ThgPhaeu | Thermacetogenium phaeum DSM 12270 | AFV11095.1 | 0 | 3 |
| Cluster II | DsfMagn8 | Desulfovibrio magneticus str. Maddingley MBC34 | ZP_11317838.1 | 3 | 3 |
| Cluster II | CloPas16 | Clostridium pasteurianum DSM 525 | ZP_20961986.1 | 0 | 3 |
| Cluster II | CloSacc6 | Clostridium saccharoperbutylacetonicum N1-4(HMT) | YP_007456102.1 | 1 | 3 |
| Cluster II | CloTerm2 | Clostridium termitidis CT1112 | EMS71272.1 | 0 | 3 |
| Cluster II | PhaFulv2 | Phaeospirillum fulvum MGU-K5 | EPY00584.1 | 1 | 3 |
| Cluster III | ChlFerro | Chlorobium ferrooxidans DSM 13031 | EAT59900.1 | 0 | 1 |
| Cluster III | UncMet11 | uncultured methanogenic archaeon RC-I | CAJ36850.1 | 0 | 3 |
| Cluster III | SnrFumar | Syntrophobacter fumaroxidans MPOB | ABK16713.1 | 1 | 1 |
| Cluster III | ChlPhaeo | Chlorobium phaeobacteroides DSM 266 | ABL64797.1 | 1 | 2 |
| Cluster III | CloTherm | Clostridium thermocellum ATCC 27405 | ABN52799.1 | 0 | 3 |
| Cluster III | DesReduc | Desulfotomaculum reducens MI-1 | ABO51325.1 | 0 | 3 |
| Cluster III | Ds0m2650 | DSM 265) | ABP37355.1 | 0 | 2 |
| Cluster III | CloKluy5 | Clostridium kluyveri DSM 555 | EDK33075.1 | 1 | 3 |
| Cluster III | CloKluy7 | Clostridium kluyveri DSM 555 | EDK35089.1 | 0 | 3 |
| Cluster III | CloBeij2 | Clostridium beijerinckii NCIMB 8052 | ABR32810.1 | 1 | 3 |
| Cluster III | CloBeij3 | Clostridium beijerinckii NCIMB 8052 | ABR34169.1 | 1 | 3 |
| Cluster III | AlkMetal | Alkaliphilus metalliredigens QYMF | ABR49651.1 | 0 | 3 |
| Cluster III | CloAceto | Clostridium acetobutylicum ATCC 824 | AAK78234.1 | 0 | 3 |
| Cluster III | CloKluy8 | Clostridium kluyveri DSM 555 | YP_001394423.1 | 1 | 3 |
| Cluster III | CloKluy9 | Clostridium kluyveri DSM 555 | YP_001394435.1 | 1 | 3 |
| Cluster III | CanMeth5 | Candidatus Methanoregula boonei 6A8 | ABS55660.1 | 0 | 3 |
| Cluster III | ChlLimi2 | Chlorobium limicola DSM 245 | ACD89768.1 | 0 | 2 |
| Cluster III | ChlPhae3 | Chlorobium phaeobacteroides BS1 | ACE04669.1 | 1 | 2 |
| Cluster III | ChrParvu | Chlorobaculum parvum NCIB 8327 | ACF12015.1 | 0 | 1 |
| Cluster III | ChhThala | Chloroherpeton thalassium ATCC 35110 | ACF13499.1 | 0 | 2 |
| Cluster III | PldPhaeo | Pelodictyon phaeoclathratiforme BU-1 | ACF44165.1 | 1 | 1 |
| Cluster III | ProAestu | Prosthecochloris aestuarii DSM 271 | ACF46645.1 | 0 | 1 |
| Cluster III | MetAcet3 | Methanosarcina acetivorans C2A | AAM07246.1 | 0 | 3 |
| Cluster III | MetMaze4 | Methanosarcina mazei Go1 | NP_632743.1 | 1 | 3 |
| Cluster III | CanAzoba | Candidatus Azobacteroides pseudotrichonymphae genomovar. CFP2 | BAG83808.1 | 0 | 3 |

| | | | | | |
|---|---|---|---|---|---|
| Cluster III | ChlTepid | Chlorobium tepidum TLS | AAM72759.1 | 0 | 1 |
| Cluster III | DscAlken | Desulfatibacillum alkenivorans AK-01 | ACL03220.1 | 0 | 0 |
| Cluster III | MhhPalu4 | Methanosphaerula palustris E1-9c | ACL15934.1 | 0 | 3 |
| Cluster III | CloKlu11 | Clostridium kluyveri NBRC 12016 | BAH05988.1 | 1 | 3 |
| Cluster III | CloKlu12 | Clostridium kluyveri NBRC 12016 | BAH06000.1 | 1 | 3 |
| Cluster III | DetAlkal | Dethiobacter alkaliphilus AHT 1 | EEG76181.1 | 0 | 1 |
| Cluster III | DesAcet3 | Desulfotomaculum acetoxidans DSM 771 | EEN16171.1 | 0 | 3 |
| Cluster III | CloButyr | Clostridium butyricum E4 str. BoNT E BL5262 | EEP56036.1 | 0 | 3 |
| Cluster III | FibSucci | Fibrobacter succinogenes subsp. succinogenes S85 | EER87422.1 | 0 | 1 |
| Cluster III | CloCell4 | Clostridium cellulovorans 743B | EES28798.1 | 1 | 3 |
| Cluster III | CloPapyr | Clostridium papyrosolvens DSM 2782 | EEU59346.1 | 1 | 3 |
| Cluster III | CloLent3 | Clostridium lentocellum DSM 5427 | EFG97044.1 | 0 | 3 |
| Cluster III | MetBark9 | Methanosarcina barkeri str. Fusaro | YP_303736.1 | 0 | 3 |
| Cluster III | AnaVari2 | Anabaena variabilis ATCC 29413 (Anabaena flos-aquae UTEX 1444) | ABA23651.1 | 1 | 3 |
| Cluster III | PldLute2 | Pelodictyon luteolum DSM 273 | ABB24385.1 | 0 | 1 |
| Cluster III | ChlChlor | Chlorobium chlorochromatii CaD3 | ABB28505.1 | 0 | 2 |
| Cluster III | DsfVulg2 | Desulfovibrio vulgaris DP4 | ABM30101.1 | 0 | 0 |
| Cluster III | MccChtho | Microcoleus chthonoplastes PCC 7420 | EDX77768.1 | 0 | 3 |
| Cluster III | VerBacte | Verrucomicrobiae bacterium DG1235 | EDY82020.1 | 0 | 0 |
| Cluster III | DsrAuto3 | Desulfobacterium autotrophicum HRM2 | ACN14079.1 | 0 | 3 |
| Cluster III | OpiBacte | Opitutaceae bacterium TAV2 | EEG22235.1 | 1 | 0 |
| Cluster III | DssThiod | Desulfonatronospira thiodismutans ASO3-1 | EEG80545.1 | 0 | 2 |
| Cluster III | DsmBacu2 | Desulfomicrobium baculatum DSM 4028 | EEK24542.1 | 0 | 0 |
| Cluster III | DsfMagne | Desulfovibrio magneticus RS-1 | BAH75547.1 | 0 | 0 |
| Cluster III | DsfSale2 | Desulfovibrio salexigens DSM 2638 | ACS78514.1 | 0 | 1 |
| Cluster III | DsfAespo | Desulfovibrio aespoeensis Aspo-2 | EFA67824.1 | 1 | 0 |
| Cluster III | EthHarbi | Ethanoligenens harbinense YUAN-3 | EFD38882.1 | 0 | 3 |
| Cluster III | RumAlbus | Ruminococcus albus 8 | EFF16860.1 | 0 | 3 |
| Cluster III | DehEthen | Dehalococcoides ethenogenes 195 | AAW39625.1 | 0 | 3 |
| Cluster III | DelProte | delta proteobacterium MLMS-1 | EAT05949.1 | 0 | 0 |
| Cluster III | DsfVulg8 | Desulfovibrio vulgaris str. 'Miyazaki F' | ACL09552.1 | 0 | 0 |
| Cluster III | DsfSpeci | Desulfovibrio sp. FW1012B | EFC22226.1 | 0 | 0 |
| Cluster III | DesRumin | Desulfotomaculum ruminis DSM 2154 | YP_004546943.1 | 0 | 3 |
| Cluster III | RumFlave | Ruminococcus flavefaciens FD-1 | ZP_06145196.1 | 0 | 3 |
| Cluster III | CorAkaji | Coraliomargarita akajimensis DSM 45221 | ADE55852.1 | 0 | 1 |
| Cluster III | ThnPote2 | Thermincola potens JR | YP_003639458.1 | 0 | 2 |
| Cluster III | DsvAlkal | Desulfurivibrio alkaliphilus AHT2 | ADH87119.1 | 0 | 1 |
| Cluster III | PreBryan | Prevotella bryantii B14 | EFI72879.1 | 0 | 3 |
| Cluster III | CloLjung | Clostridium ljungdahlii DSM 13528 | ADK13572.1 | 0 | 3 |
| Cluster III | CloLjun4 | Clostridium ljungdahlii DSM 13528 | ADK15411.1 | 1 | 3 |
| Cluster III | DelProt3 | delta proteobacterium NaphS2 | EFK11139.1 | 0 | 1 |
| Cluster III | SpiSmara | Spirochaeta smaragdinae DSM 11293 | ADK82110.1 | 1 | 3 |
| Cluster III | DlrBaars | Desulfarculus baarsii DSM 2075 | ADK83816.1 | 0 | 0 |
| Cluster III | DsfFruct | Desulfovibrio fructosovorans JJ | EFL50986.1 | 0 | 1 |
| Cluster III | DsfFruc3 | Desulfovibrio fructosovorans JJ | EFL51950.1 | 0 | 0 |
| Cluster III | AcvCellu | Acetivibrio cellulolyticus CD2 | EFL62576.1 | 0 | 3 |
| Cluster III | ThbTherm | Thermoanaerobacterium thermosaccharolyticum DSM 571 | ADL69355.1 | 0 | 3 |
| Cluster III | SpiTherm | Spirochaeta thermophila DSM 6192 | ADN01326.1 | 1 | 1 |
| Cluster III | MnpPetro | Methanoplanus petrolearius DSM 11571 | ADN35041.1 | 1 | 3 |
| Cluster III | IlyPolyt | Ilyobacter polytropus DSM 2926 | ADO83190.1 | 1 | 3 |
| Cluster III | PalProp2 | Paludibacter propionicigenes WB4 | ADQ79569.1 | 2 | 1 |
| Cluster III | DlbPropi | Desulfobulbus propionicus DSM 2032 | ADW19185.1 | 0 | 0 |
| Cluster III | DsfDesul | Desulfovibrio desulfuricans ND132 | EGB14507.1 | 0 | 0 |
| Cluster III | DesNigr2 | Desulfotomaculum nigrificans DSM 574 | EGB21515.1 | 0 | 2 |
| Cluster III | DlcAceto | Desulfobacca acetoxidans DSM 11109 | AEB08241.1 | 0 | 1 |
| Cluster III | MhtConc2 | Methanosaeta concilii GP6 | AEB67436.1 | 0 | 3 |
| Cluster III | DsfAfri2 | Desulfovibrio africanus str. Walvis Bay | EGJ49116.1 | 0 | 1 |
| Cluster III | DysGadei | Dysgonomonas gadei ATCC BAA-286 | EGK03248.1 | 1 | 2 |
| Cluster III | TreAzot4 | Treponema azotonutricium ZAS-9 | AEF80178.1 | 0 | 0 |
| Cluster III | TrePrim5 | Treponema primitia ZAS-2 | AEF83597.1 | 1 | 0 |
| Cluster III | DesCarbo | Desulfotomaculum carboxydivorans CO-1-SRB | AEF94001.1 | 0 | 2 |
| Cluster III | DsfSpec3 | Desulfovibrio sp. A2 | EGY24836.1 | 0 | 0 |
| Cluster III | DsbPostg | Desulfobacter postgatei 2ac9 | EHG07866.1 | 0 | 1 |
| Cluster III | DesGibs2 | Desulfotomaculum gibsoniae DSM 7213 | ZP_09101754.1 | 0 | 3 |
| Cluster III | CloSpe22 | Clostridium sp. DL-VIII | EHI99856.1 | 0 | 3 |
| Cluster III | CloSpe24 | Clostridium sp. DL-VIII | EHJ01844.1 | 0 | 3 |

| | | | | | |
|---|---|---|---|---|---|
| Cluster III | CloClari | Clostridium clariflavum DSM 19732 | AEV67918.1 | 0 | 3 |
| Cluster III | CloClar2 | Clostridium clariflavum DSM 19732 | AEV68734.1 | 0 | 3 |
| Cluster III | HolFoet2 | Holophaga foetida DSM 6591 | EHP11122.1 | 1 | 1 |
| Cluster III | OpiBact8 | Opitutaceae bacterium TAV1 | EHP95500.1 | 0 | 0 |
| Cluster III | OpiBac10 | Opitutaceae bacterium TAV5 | ZP_09596372.1 | 0 | 1 |
| Cluster III | CloSpe30 | Clostridium sp. BNL1100 | AEY66770.1 | 1 | 3 |
| Cluster III | DsfAfri5 | Desulfovibrio africanus str. Walvis Bay | YP_005050775.1 | 0 | 1 |
| Cluster III | TrePri15 | Treponema primitia ZAS-1 | ZP_09717077.1 | 3 | 3 |
| Cluster III | TrePri16 | Treponema primitia ZAS-1 | ZP_09718327.1 | 2 | 3 |
| Cluster III | AceWood4 | Acetobacterium woodii DSM 1030 | AFA47588.1 | 0 | 3 |
| Cluster III | FibSuc12 | Fibrobacter succinogenes subsp. succinogenes S85 | YP_005821537.1 | 0 | 2 |
| Cluster III | DsfSpec9 | Desulfovibrio sp. U5L | ZP_10077601.1 | 0 | 1 |
| Cluster III | DlmTied2 | Desulfomonile tiedjei DSM 6799 | AFM28066.1 | 1 | 2 |
| Cluster III | ThbSacc2 | Thermoanaerobacterium saccharolyticum JW/SL-YS485 | YP_006392843.1 | 0 | 3 |
| Cluster III | PlsFerm2 | Pelosinus fermentans B3 | EIW16187.1 | 1 | 3 |
| Cluster III | PlsFerm6 | Pelosinus fermentans B4 | EIW18664.1 | 0 | 3 |
| Cluster III | PlsFer33 | Pelosinus fermentans JBW45 | EIW47216.1 | 0 | 3 |
| Cluster III | CloSpe31 | Clostridium sp. MSTE9 | EJF39145.1 | 0 | 3 |
| Cluster III | MtfLimin | Methanofollis liminatans DSM 4140 | EJG06504.1 | 0 | 3 |
| Cluster III | CloArbus | Clostridium arbusti SL206 | ZP_10774019.1 | 1 | 3 |
| Cluster III | CloArbu2 | Clostridium arbusti SL206 | ZP_10774725.1 | 1 | 3 |
| Cluster III | CloArbu3 | Clostridium arbusti SL206 | ZP_10775905.1 | 1 | 3 |
| Cluster III | CloArbu4 | Clostridium arbusti SL206 | ZP_10775913.1 | 1 | 3 |
| Cluster III | CloSpe34 | Clostridium sp. Maddingley MBC34-26 | EKQ52350.1 | 0 | 3 |
| Cluster III | CloSpe36 | Clostridium sp. Maddingley MBC34-26 | EKQ53818.1 | 0 | 3 |
| Cluster III | CloSpe37 | Clostridium sp. Maddingley MBC34-26 | EKQ58112.1 | 1 | 3 |
| Cluster III | DesHydro | Desulfotomaculum hydrothermale Lam5 = DSM 18033 | CCO07505.1 | 0 | 2 |
| Cluster III | CloPas14 | Clostridium pasteurianum DSM 525 | ZP_20960283.1 | 0 | 3 |
| Cluster III | CloPas15 | Clostridium pasteurianum DSM 525 | ZP_20961374.1 | 0 | 3 |
| Cluster III | PlrSpec2 | Pleurocapsa sp. PCC 7327 | AFY79132.1 | 0 | 2 |
| Cluster III | ThbTher6 | Thermoanaerobacterium thermosaccharolyticum M0795 | YP_007299164.1 | 0 | 3 |
| Cluster III | CloPast9 | Clostridium pasteurianum DSM 525 | ELP57650.1 | 0 | 3 |
| Cluster III | CloPas10 | Clostridium pasteurianum DSM 525 | ELP57651.1 | 0 | 3 |
| Cluster III | CloPas19 | Clostridium pasteurianum DSM 525 | ELP57660.1 | 0 | 3 |
| Cluster III | CloSter2 | Clostridium stercorarium subsp. stercorarium DSM 8532 | AGC68961.1 | 0 | 3 |
| Cluster III | CloSacch | Clostridium saccharoperbutylacetonicum N1-4(HMT) | AGF55196.1 | 1 | 3 |
| Cluster III | CloSacc2 | Clostridium saccharoperbutylacetonicum N1-4(HMT) | AGF56836.1 | 0 | 3 |
| Cluster III | DsfPiezo | Desulfovibrio piezophilus C1TLV30 | CCH50130.1 | 0 | 2 |
| Cluster III | DlpSulf2 | Desulfocapsa sulfexigens DSM 10523 | YP_007466571.1 | 0 | 3 |
| Cluster III | MetMaze9 | Methanosarcina mazei Tuc01 | YP_007489420.1 | 1 | 3 |
| Cluster III | DsfAfri6 | Desulfovibrio africanus PCS | EMG37099.1 | 0 | 1 |
| Cluster III | CloTerm3 | Clostridium termitidis CT1112 | EMS71416.1 | 0 | 2 |
| Cluster III | CloPas22 | Clostridium pasteurianum BC1 | AGK98255.1 | 0 | 3 |
| Cluster III | CloPas25 | Clostridium pasteurianum BC1 | AGK98913.1 | 0 | 3 |
| Cluster III | CloPas26 | Clostridium pasteurianum BC1 | YP_007939741.1 | 0 | 3 |
| Cluster III | CloPas27 | Clostridium pasteurianum BC1 | YP_007941931.1 | 0 | 3 |
| Cluster III | CloPas29 | Clostridium pasteurianum BC1 | YP_007941941.1 | 0 | 3 |
| Cluster III | ArbSvalb | Arcticibacter svalbardensis MN12-7 | EOR94282.1 | 2 | 2 |
| Cluster III | LchBact5 | Lachnospiraceae bacterium 3-1 | EOS26764.1 | 0 | 3 |
| Cluster III | CloPapy5 | Clostridium papyrosolvens C7 | EPR11926.1 | 1 | 3 |
| Cluster III | DsfAlkal | Desulfovibrio alkalitolerans DSM 16529 | EPR32344.1 | 0 | 0 |
| Cluster III | DsfSpe11 | Desulfovibrio sp. X2 | EPR42537.1 | 0 | 1 |
| Cluster III | SpoOvat2 | Sporomusa ovata DSM 2662 | EQB27148.1 | 0 | 3 |
| Cluster IV | MhcLabre | Methanocorpusculum labreanum Z | ABN07210.1 | 2 | 2 |
| Cluster IV | CloKluyv | Clostridium kluyveri DSM 555 | EDK34473.1 | 1 | 3 |
| Cluster IV | CloBotul | Clostridium botulinum A str. ATCC 3502 | CAL82242.1 | 1 | 3 |
| Cluster IV | CloBotu9 | Clostridium botulinum NCTC 2916 | ZP_02612730.1 | 2 | 3 |
| Cluster IV | CloBot10 | Clostridium botulinum Bf | ZP_02618040.1 | 2 | 3 |
| Cluster IV | CloBot11 | Clostridium botulinum B1 str. Okra | ACA43986.1 | 2 | 3 |
| Cluster IV | CloBot14 | Clostridium botulinum A3 str. Loch Maree | YP_001786042.1 | 0 | 3 |
| Cluster IV | CloSporo | Clostridium sporogenes ATCC 15579 | EDU38536.1 | 2 | 3 |
| Cluster IV | DstHafni | Desulfitobacterium hafniense Y51 | BAE85358.1 | 0 | 2 |
| Cluster IV | RhdPalus | Rhodopseudomonas palustris DX-1 | EFC25942.1 | 0 | 1 |
| Cluster IV | DstHafn3 | Desulfitobacterium hafniense DCB-2 | ACL19859.1 | 0 | 2 |
| Cluster IV | CopComes | Coprococcus comes ATCC 27758 | EEG91327.1 | 1 | 2 |
| Cluster IV | SntWolfe | Syntrophomonas wolfei subsp. wolfei str. Goettingen | ABI67746.1 | 1 | 3 |

| | | | | | |
|---|---|---|---|---|---|
| Cluster IV | AlkOreml | Alkaliphilus oremlandii OhILAs | ABW17872.1 | 1 | 3 |
| Cluster IV | CloBolte | Clostridium bolteae ATCC BAA-613 | EDP17532.1 | 2 | 3 |
| Cluster IV | DorFormi | Dorea formicigenerans ATCC 27755 | EDR47285.1 | 3 | 3 |
| Cluster IV | CloLento | Clostridium lentocellum DSM 5427 | EFG96608.1 | 0 | 3 |
| Cluster IV | GeoUrani | Geobacter uraniireducens Rf4 | ABQ28038.1 | 3 | 3 |
| Cluster IV | MhtTherm | Methanosaeta thermophila PT (Methanothrix thermophila PT) | ABK14960.1 | 3 | 3 |
| Cluster IV | MhpStadt | Methanosphaera stadtmanae DSM 3091 | ABC57502.1 | 3 | 3 |
| Cluster IV | MtdInfer | Methanocaldococcus infernus ME | EER82097.1 | 3 | 3 |
| Cluster IV | MhdBurto | Methanococcoides burtonii DSM 6242 | ABE51973.1 | 3 | 3 |
| Cluster IV | MhnVann2 | Methanococcus vannielii SB | ABR55051.1 | 1 | 3 |
| Cluster IV | MhrKandl | Methanopyrus kandleri AV19 | AAM02629.1 | 2 | 3 |
| Cluster IV | MetAceti | Methanosarcina acetivorans C2A | AAM06982.1 | 3 | 3 |
| Cluster IV | MetMazei | Methanosarcina mazei Go1 | AAM30210.1 | 3 | 3 |
| Cluster IV | MhhPalus | Methanosphaerula palustris E1-9c | ACL15489.1 | 3 | 3 |
| Cluster IV | MetBark5 | Methanosarcina barkeri str. Fusaro | AAZ69331.1 | 3 | 3 |
| Cluster IV | MhcLabr3 | Methanocorpusculum labreanum Z | ABN06341.1 | 1 | 3 |
| Cluster IV | MtrSmith | Methanobrevibacter smithii ATCC 35061 | ABQ87912.1 | 3 | 3 |
| Cluster IV | RhdPalu3 | Rhodopseudomonas palustris BisA53 | ABJ07125.1 | 1 | 3 |
| Cluster IV | CanMetha | Candidatus Methanoregula boonei 6A8 | ABS56522.1 | 2 | 3 |
| Cluster IV | RhdPalu6 | Rhodopseudomonas palustris TIE-1 | ACF01415.1 | 1 | 3 |
| Cluster IV | RhdPalu7 | Rhodopseudomonas palustris TIE-1 | YP_001991585.1 | 1 | 3 |
| Cluster IV | DstHafn5 | Desulfitobacterium hafniense DCB-2 | YP_002457845.1 | 2 | 3 |
| Cluster IV | GeoSpeci | Geobacter sp. FRC-32 | ACM20811.1 | 2 | 3 |
| Cluster IV | CloCell2 | Clostridium cellulovorans 743B | EES29132.1 | 2 | 3 |
| Cluster IV | RhdPalu9 | Rhodopseudomonas palustris CGA009 | CAE27794.1 | 2 | 3 |
| Cluster IV | RhsRubru | Rhodospirillum rubrum ATCC 11170 | ABC21599.1 | 1 | 3 |
| Cluster IV | DstHafn6 | Desulfitobacterium hafniense Y51 | BAE85800.1 | 2 | 3 |
| Cluster IV | UncMeth9 | uncultured methanogenic archaeon RC-I | CAJ37158.1 | 3 | 3 |
| Cluster IV | RhbSphae | Rhodobacter sphaeroides ATCC 17025 | ABP69680.1 | 1 | 3 |
| Cluster IV | DstHafn8 | Desulfitobacterium hafniense Y51 | BAE85651.1 | 1 | 3 |
| Cluster IV | CanMeth3 | Candidatus Methanoregula boonei 6A8 | ABS56597.1 | 0 | 3 |
| Cluster IV | DstHaf11 | Desulfitobacterium hafniense Y51 | YP_516595.1 | 2 | 3 |
| Cluster IV | SelFlueg | Selenomonas flueggei ATCC 43531 | EEQ48627.1 | 1 | 3 |
| Cluster IV | DesAceto | Desulfotomaculum acetoxidans DSM 771 | EEN15621.1 | 2 | 3 |
| Cluster IV | MncMaris | Methanoculleus marisnigri JR1 | ABN57863.1 | 2 | 3 |
| Cluster IV | RhdPal13 | Rhodopseudomonas palustris TIE-1 | ACF01394.1 | 1 | 3 |
| Cluster IV | MhhPalu3 | Methanosphaerula palustris E1-9c | ACL15621.1 | 0 | 3 |
| Cluster IV | DsrAutot | Desulfobacterium autotrophicum HRM2 | ACN13985.1 | 2 | 3 |
| Cluster IV | MhmOkina | Methanothermococcus okinawensis IH1 | EFL49005.1 | 0 | 3 |
| Cluster IV | MnsHunga | Methanospirillum hungatei JF-1 | ABD40545.1 | 0 | 3 |
| Cluster IV | RsfSpec2 | Roseiflexus sp. RS-1 | YP_001275558.1 | 1 | 3 |
| Cluster IV | MhnAeoli | Methanococcus aeolicus Nankai-3 | ABR56800.1 | 0 | 3 |
| Cluster IV | MtdJanna | Methanocaldococcus jannaschii DSM 2661 | NP_247874.1 | 0 | 3 |
| Cluster IV | RsfCast2 | Roseiflexus castenholzii DSM 13941 | YP_001434094.1 | 0 | 3 |
| Cluster IV | MhnVolta | Methanococcus voltae A3 | EDP41050.1 | 1 | 3 |
| Cluster IV | MtdFerve | Methanocaldococcus fervens AG86 | EER76341.1 | 2 | 3 |
| Cluster IV | MhnAeol3 | Methanococcus aeolicus Nankai-3 | ABR56092.1 | 1 | 3 |
| Cluster IV | MhnMari4 | Methanococcus maripaludis C7 | ABR66213.1 | 1 | 3 |
| Cluster IV | MhnMari7 | Methanococcus maripaludis C6 | ABX01620.1 | 1 | 3 |
| Cluster IV | MhnMar13 | Methanococcus maripaludis S2 | NP_987267.1 | 1 | 3 |
| Cluster IV | MetAcet9 | Methanosarcina acetivorans C2A | AAM05043.1 | 2 | 3 |
| Cluster IV | MhnMar15 | Methanococcus maripaludis C5 | ABO35825.1 | 1 | 3 |
| Cluster IV | DorLongi | Dorea longicatena DSM 13814 | EDM62024.1 | 2 | 3 |
| Cluster IV | CloBartl | Clostridium bartlettii DSM 16795 | EDQ96257.1 | 1 | 3 |
| Cluster IV | CopCome3 | Coprococcus comes ATCC 27758 | EEG91338.1 | 3 | 3 |
| Cluster IV | RumAlbu5 | Ruminococcus albus 8 | EFF17186.1 | 3 | 3 |
| Cluster IV | EubHalli | Eubacterium hallii DSM 3353 | EEG38057.1 | 1 | 3 |
| Cluster IV | DorForm3 | Dorea formicigenerans ATCC 27755 | EDR47172.1 | 1 | 3 |
| Cluster IV | HydroDSM | hydrogenotrophicus DSM 10507) | EEG50047.1 | 2 | 3 |
| Cluster IV | RumAlbu7 | Ruminococcus albus 7 | EFM00075.1 | 2 | 3 |
| Cluster IV | DorLong3 | Dorea longicatena DSM 13814 | EDM62016.1 | 1 | 3 |
| Cluster IV | RumObeum | Ruminococcus obeum ATCC 29174 | EDM89181.1 | 1 | 3 |
| Cluster IV | DstHaf16 | Desulfitobacterium hafniense Y51 | BAE85627.1 | 1 | 3 |
| Cluster IV | CloBolt3 | Clostridium bolteae ATCC BAA-613 | EDP15245.1 | 1 | 3 |
| Cluster IV | ClsBacte | Clostridiales bacterium 1_7_47FAA | EEQ59017.1 | 1 | 3 |
| Cluster IV | RumAlbu9 | Ruminococcus albus 8 | EFF17189.1 | 1 | 3 |

| | | | | | |
|---|---|---|---|---|---|
| Cluster IV | EubHall3 | Eubacterium hallii DSM 3353 | EEG38049.1 | 3 | 3 |
| Cluster IV | DorForm5 | Dorea formicigenerans ATCC 27755 | EDR47181.1 | 3 | 3 |
| Cluster IV | RumAlb11 | Ruminococcus albus 7 | EFM00078.1 | 3 | 3 |
| Cluster IV | RumObeu3 | Ruminococcus obeum ATCC 29174 | EDM89190.1 | 3 | 3 |
| Cluster IV | DstHaf18 | Desulfitobacterium hafniense DCB-2 | ACL19588.1 | 1 | 3 |
| Cluster IV | FusNucle | Fusobacterium nucleatum subsp. nucleatum ATCC 25586 | NP_603210.1 | 3 | 3 |
| Cluster IV | MetAce11 | Methanosarcina acetivorans C2A | AAM05435.1 | 2 | 3 |
| Cluster IV | FusUlcer | Fusobacterium ulcerans ATCC 49185 | ZP_05633122.1 | 1 | 3 |
| Cluster IV | MnhMahii | Methanohalophilus mahii DSM 5219 | ADE36911.1 | 3 | 3 |
| Cluster IV | CloCarbo | Clostridium carboxidivorans P7 | EET88725.1 | 3 | 3 |
| Cluster IV | CloCarb2 | Clostridium carboxidivorans P7 | EET88732.1 | 2 | 3 |
| Cluster IV | CloDiffi | Clostridium difficile QCD-23m63 | ZP_05400456.1 | 1 | 3 |
| Cluster IV | MitMulta | Mitsuokella multacida DSM 20544 | ZP_05403609.1 | 2 | 3 |
| Cluster IV | DesAcet5 | Desulfotomaculum acetoxidans DSM 771 | ACV61347.1 | 2 | 3 |
| Cluster IV | DesAcet7 | Desulfotomaculum acetoxidans DSM 771 | YP_003189970.1 | 2 | 3 |
| Cluster IV | DiaInvis | Dialister invisus DSM 15470 | ZP_05732897.1 | 0 | 3 |
| Cluster IV | FusSpeci | Fusobacterium sp. 3_1_33 | EEW93992.1 | 3 | 3 |
| Cluster IV | SelSputi | Selenomonas sputigena ATCC 35185 | EEX77752.1 | 1 | 3 |
| Cluster IV | MtdVulca | Methanocaldococcus vulcanius M7 | ACX72742.1 | 1 | 3 |
| Cluster IV | UncArc82 | uncultured archaeon | CBH36741.1 | 0 | 3 |
| Cluster IV | UncArc83 | uncultured archaeon | CBH39757.1 | 0 | 3 |
| Cluster IV | UncArc84 | uncultured archaeon | CBH40055.1 | 0 | 3 |
| Cluster IV | SlaExigu | Slackia exigua ATCC 700122 | EEZ60752.1 | 2 | 3 |
| Cluster IV | MnlPalud | Methanocella paludicola SANAE | BAI60436.1 | 1 | 3 |
| Cluster IV | MnlPalu2 | Methanocella paludicola SANAE | BAI60977.1 | 3 | 3 |
| Cluster IV | PyrPisco | Pyramidobacter piscolens W5455 | EFB90680.1 | 0 | 2 |
| Cluster IV | MtrRumi2 | Methanobrevibacter ruminantium M1 | ADC47316.1 | 3 | 3 |
| Cluster IV | MtrSmit9 | Methanobrevibacter smithii DSM 2374 | EFC93706.1 | 3 | 3 |
| Cluster IV | MtdSpec2 | Methanocaldococcus sp. FS406-22 | ADC69956.1 | 0 | 3 |
| Cluster IV | ClsGenom | Clostridiales genomosp. BVAB3 str. UPII9-5 | ADC90429.1 | 0 | 3 |
| Cluster IV | MegGenom | Megasphaera genomosp. type_1 str. 28L | EFD94373.1 | 1 | 3 |
| Cluster IV | MegGeno2 | Megasphaera genomosp. type_1 str. 28L | EFD94380.1 | 3 | 3 |
| Cluster IV | ButFibri | Butyrivibrio fibrisolvens 16/4 | CBK74006.1 | 2 | 3 |
| Cluster IV | CopCatus | Coprococcus catus GD/7 | CBK81299.1 | 3 | 3 |
| Cluster IV | CopCatu2 | Coprococcus catus GD/7 | CBK81306.1 | 1 | 3 |
| Cluster IV | RumTorqu | Ruminococcus torques L2-14 | CBL25132.1 | 3 | 3 |
| Cluster IV | RumTorq2 | Ruminococcus torques L2-14 | CBL25139.1 | 1 | 3 |
| Cluster IV | TurSangu | Turicibacter sanguinis PC909 | EFF64704.1 | 1 | 3 |
| Cluster IV | SelNoxia | Selenomonas noxia ATCC 43541 | EFF66827.1 | 2 | 3 |
| Cluster IV | RhbCaps7 | Rhodobacter capsulatus SB 1003 | ADE85966.1 | 1 | 3 |
| Cluster IV | FusSpec3 | Fusobacterium sp. 3_1_27 | EFG34074.1 | 3 | 3 |
| Cluster IV | GorPamel | Gordonibacter pamelaeae 7-10-1-b | CBL04240.1 | 2 | 2 |
| Cluster IV | RumObeu5 | Ruminococcus obeum A2-162 | CBL23059.1 | 3 | 3 |
| Cluster IV | RumObeu6 | Ruminococcus obeum A2-162 | CBL23067.1 | 1 | 3 |
| Cluster IV | CloBot21 | Clostridium botulinum F str. 230613 | ADF98499.1 | 2 | 3 |
| Cluster IV | FusNucl4 | Fusobacterium nucleatum subsp. nucleatum ATCC 23726 | ZP_06869933.1 | 3 | 3 |
| Cluster IV | MnbEvest | Methanohalobium evestigatum Z-7303 | ADI73466.1 | 3 | 3 |
| Cluster IV | CloLjun2 | Clostridium ljungdahlii DSM 13528 | ADK15363.1 | 2 | 3 |
| Cluster IV | CloLjun3 | Clostridium ljungdahlii DSM 13528 | ADK15370.1 | 3 | 3 |
| Cluster IV | DsfFruc2 | Desulfovibrio fructosovorans JJ | EFL51485.1 | 2 | 3 |
| Cluster IV | MtnMarb4 | Methanothermobacter marburgensis str. Marburg | YP_003849930.1 | 1 | 3 |
| Cluster IV | SelSpeci | Selenomonas sp. oral taxon 149 str. 67H29BP | EFM23483.1 | 2 | 3 |
| Cluster IV | MnpPetr3 | Methanoplanus petrolearius DSM 11571 | ADN37126.1 | 0 | 3 |
| Cluster IV | MnpPetr5 | Methanoplanus petrolearius DSM 11571 | YP_003895423.1 | 2 | 2 |
| Cluster IV | EubLimos | Eubacterium limosum KIST612 | ADO36703.1 | 1 | 3 |
| Cluster IV | OslTric5 | Oscillochloris trichoides DG-6 | ZP_07684114.1 | 1 | 3 |
| Cluster IV | MegMicro | Megasphaera micronuciformis F0359 | EFQ04981.1 | 3 | 3 |
| Cluster IV | MegMicr2 | Megasphaera micronuciformis F0359 | EFQ04990.1 | 3 | 3 |
| Cluster IV | RhmVann3 | Rhodomicrobium vannielii ATCC 17100 | ADP72734.1 | 2 | 3 |
| Cluster IV | RhmVann4 | Rhodomicrobium vannielii ATCC 17100 | ADP72762.1 | 1 | 3 |
| Cluster IV | MntFervi | Methanothermus fervidus DSM 2088 | ADP77182.1 | 3 | 3 |
| Cluster IV | EubCellu | Eubacterium cellulosolvens 6 | EFR64597.1 | 2 | 3 |
| Cluster IV | SelSpec4 | Selenomonas sp. oral taxon 137 str. F0430 | ZP_07828878.1 | 2 | 3 |
| Cluster IV | PsrAlac2 | Pseudoramibacter alactolyticus ATCC 23263 | EFV02746.1 | 1 | 2 |
| Cluster IV | PsrAlac3 | Pseudoramibacter alactolyticus ATCC 23263 | ZP_07920173.1 | 2 | 2 |
| Cluster IV | SelArte2 | Selenomonas artemidis F0399 | ZP_08030990.1 | 1 | 3 |

| | | | | | |
|---|---|---|---|---|---|
| Cluster IV | PhsSpec2 | Phascolarctobacterium sp. YIT 12067 | ZP_08076835.1 | 1 | 3 |
| Cluster IV | SnbGlyco | Syntrophobotulus glycolicus DSM 8271 | ADY57074.1 | 2 | 3 |
| Cluster IV | CloSpe18 | Clostridium sp. D5 | ZP_08130083.1 | 2 | 3 |
| Cluster IV | MhbSpeci | Methanobacterium sp. AL-21 | ADZ09424.1 | 1 | 3 |
| Cluster IV | MhbSpec3 | Methanobacterium sp. AL-21 | ADZ10312.1 | 3 | 3 |
| Cluster IV | MhtConc3 | Methanosaeta concilii GP6 | YP_004383166.1 | 3 | 3 |
| Cluster IV | ThdNaru2 | Thermodesulfobium narugense DSM 14796 | YP_004438257.1 | 2 | 3 |
| Cluster IV | TreBren4 | Treponema brennaborense DSM 12168 | YP_004439781.1 | 0 | 3 |
| Cluster IV | TreAzot3 | Treponema azotonutricium ZAS-9 | AEF80058.1 | 3 | 3 |
| Cluster IV | TreAzot6 | Treponema azotonutricium ZAS-9 | AEF83461.1 | 2 | 3 |
| Cluster IV | TrePrim6 | Treponema primitia ZAS-2 | AEF84422.1 | 0 | 2 |
| Cluster IV | TrePrim7 | Treponema primitia ZAS-2 | AEF84719.1 | 1 | 3 |
| Cluster IV | TrePrim8 | Treponema primitia ZAS-2 | AEF85643.1 | 3 | 3 |
| Cluster IV | MnrIgneu | Methanotorris igneus Kol 5 | AEF96745.1 | 1 | 3 |
| Cluster IV | MnrIgne2 | Methanotorris igneus Kol 5 | AEF96954.1 | 2 | 3 |
| Cluster IV | MegSpeci | Megasphaera sp. UPII 199-6 | EGL40472.1 | 3 | 3 |
| Cluster IV | DesKuzn2 | Desulfotomaculum kuznetsovii DSM 6115 | AEG15744.1 | 3 | 3 |
| Cluster IV | MhbSpec8 | Methanobacterium sp. SWAN-1 | AEG18043.1 | 1 | 3 |
| Cluster IV | DesKuzn4 | Desulfotomaculum kuznetsovii DSM 6115 | YP_004517541.1 | 0 | 3 |
| Cluster IV | MhbSpe10 | Methanobacterium sp. SWAN-1 | YP_004519382.1 | 3 | 3 |
| Cluster IV | TreAzot8 | Treponema azotonutricium ZAS-9 | YP_004526190.1 | 1 | 3 |
| Cluster IV | CenPeri2 | Centipeda periodontii DSM 2778 | ZP_08502394.1 | 1 | 3 |
| Cluster IV | MnmZhili | Methanosalsum zhilinae DSM 4017 | AEH60342.1 | 3 | 3 |
| Cluster IV | FusSpec6 | Fusobacterium sp. 11_3_2 | ZP_08599234.1 | 3 | 3 |
| Cluster IV | AcnLong3 | Acetonema longum DSM 6540 | EGO62663.1 | 0 | 2 |
| Cluster IV | AcnLong4 | Acetonema longum DSM 6540 | EGO64491.1 | 2 | 3 |
| Cluster IV | CloSpe19 | Clostridium sp. SY8519 | BAK47519.1 | 3 | 3 |
| Cluster IV | MhnMar17 | Methanococcus maripaludis X1 | AEK19038.1 | 1 | 3 |
| Cluster IV | PepSpec2 | Peptoniphilus sp. oral taxon 375 str. F0436 | EGS31172.1 | 2 | 3 |
| Cluster IV | MegSpec5 | Megasphaera sp. UPII 135-E | EGS32132.1 | 1 | 3 |
| Cluster IV | MegSpec6 | Megasphaera sp. UPII 135-E | EGS32192.1 | 3 | 3 |
| Cluster IV | MegElsde | Megasphaera elsdenii DSM 20460 | CCC72668.1 | 3 | 2 |
| Cluster IV | PepSpec3 | Peptoniphilus sp. oral taxon 375 str. F0436 | ZP_08708941.1 | 3 | 3 |
| Cluster IV | DorForm8 | Dorea formicigenerans 4_6_53AFAA | EGX77950.1 | 1 | 3 |
| Cluster IV | DorForm9 | Dorea formicigenerans 4_6_53AFAA | ZP_08848298.1 | 3 | 3 |
| Cluster IV | MegElsd4 | Megasphaera elsdenii DSM 20460 | YP_004765504.1 | 2 | 3 |
| Cluster IV | CloCitro | Clostridium citroniae WAL-17108 | EHE97178.1 | 1 | 3 |
| Cluster IV | CloCitr2 | Clostridium citroniae WAL-17108 | EHE99575.1 | 1 | 3 |
| Cluster IV | SelInfel | Selenomonas infelix ATCC 43532 | EHG18873.1 | 2 | 3 |
| Cluster IV | FusNucl7 | Fusobacterium nucleatum subsp. polymorphum F0401 | EHG19730.1 | 3 | 3 |
| Cluster IV | SelNoxi3 | Selenomonas noxia F0398 | EHG23561.1 | 2 | 3 |
| Cluster IV | MnnTard2 | Methanolinea tarda NOBI-1 | ZP_09043736.1 | 1 | 3 |
| Cluster IV | CloSpe25 | Clostridium sp. DL-VIII | ZP_09202603.1 | 2 | 3 |
| Cluster IV | CloClar3 | Clostridium clariflavum DSM 19732 | AEV68879.1 | 2 | 3 |
| Cluster IV | DstHaf21 | Desulfitobacterium hafniense DP7 | EHL03953.1 | 2 | 3 |
| Cluster IV | DstHaf23 | Desulfitobacterium hafniense DP7 | EHL06727.1 | 1 | 3 |
| Cluster IV | DstHaf24 | Desulfitobacterium hafniense DP7 | EHL06770.1 | 1 | 3 |
| Cluster IV | DstHaf25 | Desulfitobacterium hafniense DP7 | EHL08017.1 | 2 | 3 |
| Cluster IV | DstHaf26 | Desulfitobacterium hafniense DP7 | EHL08463.1 | 0 | 2 |
| Cluster IV | SubSpeci | Subdoligranulum sp. 4_3_54A2FAA | EHL67834.1 | 3 | 3 |
| Cluster IV | AngGemin | Anaeroglobus geminatus F0357 | EHM39130.1 | 1 | 3 |
| Cluster IV | FlaPlau2 | Flavonifractor plautii ATCC 29863 | EHM51834.1 | 2 | 3 |
| Cluster IV | SngSpec2 | Synergistes sp. 3_1_syn1 | ZP_09363008.1 | 0 | 2 |
| Cluster IV | FlaPlau4 | Flavonifractor plautii ATCC 29863 | ZP_09385536.1 | 2 | 3 |
| Cluster IV | CloSpor3 | Clostridium sporogenes PA3679 | EHN15278.1 | 2 | 3 |
| Cluster IV | LchBacte | Lachnospiraceae bacterium ACC2 | EHO16524.1 | 0 | 2 |
| Cluster IV | FusNucl8 | Fusobacterium nucleatum subsp. animalis OT 420 | EHO78329.1 | 3 | 3 |
| Cluster IV | EubInfir | Eubacterium infirmum F0142 | EHO85899.1 | 2 | 3 |
| Cluster IV | LchBact4 | Lachnospiraceae bacterium 7_1_58FAA | ZP_09530543.1 | 2 | 3 |
| Cluster IV | DiaSucc2 | Dialister succinatiphilus YIT 11850 | ZP_09546168.1 | 0 | 3 |
| Cluster IV | HolFoet4 | Holophaga foetida DSM 6591 | ZP_09579131.1 | 3 | 3 |
| Cluster IV | MnrForm2 | Methanotorris formicicus Mc-S-70 | EHP85405.1 | 0 | 3 |
| Cluster IV | DslYoun3 | Desulfosporosinus youngiae DSM 17734 | ZP_09653472.1 | 3 | 3 |
| Cluster IV | MnpLimi2 | Methanoplanus limicola DSM 2279 | ZP_09703043.1 | 0 | 3 |
| Cluster IV | TrePri14 | Treponema primitia ZAS-1 | ZP_09716922.1 | 2 | 3 |
| Cluster IV | TrePri17 | Treponema primitia ZAS-1 | ZP_09718583.1 | 2 | 3 |

| | | | | | |
|---|---|---|---|---|---|
| Cluster IV | AceWood2 | Acetobacterium woodii DSM 1030 | AFA46824.1 | 2 | 3 |
| Cluster IV | MhtHaru2 | Methanosaeta harundinacea 6Ac | YP_005919063.1 | 3 | 3 |
| Cluster IV | CloBot23 | Clostridium botulinum H04402 065 | YP_005677318.1 | 0 | 3 |
| Cluster IV | EubCell5 | Eubacterium cellulosolvens 6 | EIM56563.1 | 1 | 3 |
| Cluster IV | DstDeha4 | Desulfitobacterium dehalogenans ATCC 51507 | AFL99851.1 | 1 | 3 |
| Cluster IV | PlsFer15 | Pelosinus fermentans A11 | EIW26696.1 | 0 | 2 |
| Cluster IV | PlsFer22 | Pelosinus fermentans B3 | EIW31027.1 | 2 | 3 |
| Cluster IV | PlsFer32 | Pelosinus fermentans JBW45 | EIW46536.1 | 0 | 2 |
| Cluster IV | PlsFer34 | Pelosinus fermentans JBW45 | EIW49245.1 | 1 | 3 |
| Cluster IV | PlsFer40 | Pelosinus fermentans DSM 17108 | ZP_10327603.1 | 1 | 3 |
| Cluster IV | CloSpe32 | Clostridium sp. MSTE9 | EJF39147.1 | 1 | 3 |
| Cluster IV | CloSpe33 | Clostridium sp. MSTE9 | EJF40737.1 | 0 | 3 |
| Cluster IV | MtfLimi2 | Methanofollis liminatans DSM 4140 | EJG07797.1 | 2 | 3 |
| Cluster IV | MncBour2 | Methanoculleus bourgensis MS2 | YP_006543967.1 | 1 | 3 |
| Cluster IV | SelSpec7 | Selenomonas sp. FOBRC6 | ZP_10804954.1 | 2 | 3 |
| Cluster IV | MogSpec2 | Mogibacterium sp. CM50 | EJU22321.1 | 1 | 3 |
| Cluster IV | SelSpec8 | Selenomonas sp. CM52 | EJU27856.1 | 1 | 3 |
| Cluster IV | MogSpec3 | Mogibacterium sp. CM50 | ZP_10884381.1 | 3 | 3 |
| Cluster IV | CloAcid2 | Clostridium acidurici 9a | AFS79164.1 | 2 | 3 |
| Cluster IV | CloAcid3 | Clostridium acidurici 9a | YP_006787290.1 | 0 | 3 |
| Cluster IV | MhbForm4 | Methanobacterium formicicum DSM 3637 | ZP_11179328.1 | 3 | 3 |
| Cluster IV | BacAzot2 | Bacillus azotoformans LMG 9581 | ZP_11311565.1 | 3 | 3 |
| Cluster IV | FusNuc10 | Fusobacterium nucleatum ChDC F128 | ZP_15973518.1 | 3 | 3 |
| Cluster IV | FusPeri2 | Fusobacterium periodonticum D10 | ZP_16397208.1 | 3 | 3 |
| Cluster IV | SutWads2 | Sutterella wadsworthensis 2_1_59BFAA | ZP_16429155.1 | 0 | 3 |
| Cluster IV | FusNuc14 | Fusobacterium nucleatum subsp. animalis F0419 | ZP_17124845.1 | 3 | 3 |
| Cluster IV | VeiRatti | Veillonella ratti ACS-216-V-Col6b | EKU77481.1 | 2 | 3 |
| Cluster IV | ThpArcha | Thermoplasmatales archaeon BRNA1 | AGI48130.1 | 2 | 3 |
| Cluster IV | CanMeth7 | Candidatus Methanomethylophilus alvus Mx1201 | AGI85899.1 | 3 | 3 |
| Cluster IV | MncSpeci | Methanoculleus sp. CAG:1088 | CDF30912.1 | 3 | 3 |
| Cluster IV | MtnTher3 | Methanothermobacter thermautotrophicus CaT2 | BAM69819.1 | 3 | 3 |
| Cluster IV | MtgForm2 | Methanoregula formicicum SMSP | YP_007248338.1 | 2 | 3 |
| Cluster IV | ThbTher5 | Thermoanaerobacterium thermosaccharolyticum M0795 | YP_007298918.1 | 2 | 3 |
| Cluster IV | MetMaze6 | Methanosarcina mazei Tuc01 | AGF95957.1 | 3 | 3 |
| Cluster IV | FusNuc15 | Fusobacterium nucleatum CC53 | EMP17139.1 | 3 | 3 |
| Cluster IV | ThpArch2 | Thermoplasmatales archaeon BRNA1 | AGI48239.1 | 0 | 2 |
| Cluster IV | CloTermi | Clostridium termitidis CT1112 | EMS71146.1 | 3 | 3 |
| Cluster IV | CanMeth8 | Candidatus Methanomethylophilus alvus Mx1201 | AGI86016.1 | 3 | 3 |
| Cluster IV | MtdVillo | Methanocaldococcus villosus KIN24-T80 | ENN96061.1 | 1 | 3 |
| Cluster IV | CanMet12 | Candidatus Methanomethylophilus alvus Mx1201 | YP_007713978.1 | 0 | 3 |
| Cluster IV | CloClos3 | Clostridium clostridioforme 90A7 | ENZ16783.1 | 1 | 3 |
| Cluster IV | CloClos4 | Clostridium clostridioforme 90A3 | ENZ21722.1 | 3 | 3 |
| Cluster IV | CloBolt5 | Clostridium bolteae 90B3 | ENZ39210.1 | 1 | 3 |
| Cluster IV | CloPas24 | Clostridium pasteurianum BC1 | AGK98882.1 | 3 | 3 |
| Cluster IV | DorSpec2 | Dorea sp. 5-2 | EOS81066.1 | 3 | 3 |
| Cluster IV | MmsSpec2 | Methanomassiliicoccus sp. Mx1-Issoire | AGN26900.1 | 3 | 3 |
| Cluster IV | MmsSpec3 | Methanomassiliicoccus sp. Mx1-Issoire | AGN27014.1 | 3 | 3 |
| Cluster IV | MtrSpec2 | Methanobrevibacter sp. AbM4 | YP_008075069.1 | 3 | 3 |
| Cluster IV | MmsSpec4 | Candidatus Methanomassiliicoccus intestinalis Issoire-Mx1 | YP_008071420.1 | 3 | 3 |
| Cluster IV | TreSocra | Treponema socranskii subsp. paredis ATCC 35535 | EPF27457.1 | 0 | 3 |
| Cluster IV | TreMalto | Treponema maltophilum ATCC 51939 | EPF31284.1 | 0 | 3 |
| Cluster IV | MegSpe11 | Megasphaera sp. NM10 | EPP18223.1 | 2 | 3 |
| Cluster IV | MegSpe12 | Megasphaera sp. NM10 | EPP18231.1 | 3 | 3 |
| Cluster IV | CloPapy6 | Clostridium papyrosolvens C7 | EPR13521.1 | 2 | 3 |
| Cluster IV | EubSpeci | Eubacterium sp. CAG:146 | CCY14113.1 | 1 | 3 |
| Cluster IV | EubSpec2 | Eubacterium sp. CAG:146 | CCY14121.1 | 3 | 3 |
| Cluster IV | RumSpec7 | Ruminococcus sp. CAG:60 | CCY31912.1 | 2 | 3 |
| Cluster IV | RumSpec8 | Ruminococcus sp. CAG:17 | CCY97526.1 | 1 | 3 |
| Cluster IV | RumSpec9 | Ruminococcus sp. CAG:17 | CCY97534.1 | 2 | 3 |
| Cluster IV | RsbSpeci | Roseburia sp. CAG:50 | CCZ64898.1 | 3 | 3 |
| Cluster IV | RsbSpec2 | Roseburia sp. CAG:50 | CCZ64907.1 | 1 | 3 |
| Cluster IV | RsbSpec3 | Roseburia sp. CAG:18 | CCZ78924.1 | 1 | 3 |
| Cluster IV | RsbSpec4 | Roseburia sp. CAG:18 | CCZ78932.1 | 3 | 3 |
| Cluster IV | CloBart3 | Clostridium bartlettii CAG:1329 | CDA10086.1 | 1 | 3 |
| Cluster IV | CloBart4 | Clostridium bartlettii CAG:1329 | CDA09285.1 | 1 | 3 |
| Cluster IV | FirBacte | Firmicutes bacterium CAG:536 | CDA34612.1 | 2 | 3 |

| | | | | | |
|---|---|---|---|---|---|
| Cluster IV | FirBact2 | Firmicutes bacterium CAG:536 | CDA34619.1 | 1 | 3 |
| Cluster IV | FirBact3 | Firmicutes bacterium CAG:145 | CDB02908.1 | 2 | 3 |
| Cluster IV | EubHall5 | Eubacterium hallii CAG:12 | CDB19156.1 | 1 | 3 |
| Cluster IV | PhsSpec3 | Phascolarctobacterium sp. CAG:266 | CDB34630.1 | 0 | 2 |
| Cluster IV | PhsSpec4 | Phascolarctobacterium sp. CAG:207 | CDB45816.1 | 0 | 3 |
| Cluster IV | BlaSpeci | Blautia sp. CAG:237 | CDB78085.1 | 1 | 3 |
| Cluster IV | BlaSpec2 | Blautia sp. CAG:237 | CDB78094.1 | 3 | 3 |
| Cluster IV | CopCome5 | Coprococcus comes CAG:19 | CDB84421.1 | 3 | 3 |
| Cluster IV | RumSpe10 | Ruminococcus sp. CAG:57 | CDC67214.1 | 3 | 3 |
| Cluster IV | RumSpe11 | Ruminococcus sp. CAG:57 | CDC67217.1 | 2 | 3 |
| Cluster IV | PhsSucci | Phascolarctobacterium succinatutens CAG:287 | CDD12711.1 | 0 | 3 |
| Cluster IV | PhsSucc2 | Phascolarctobacterium succinatutens CAG:287 | CDD10777.1 | 1 | 3 |
| Cluster IV | FirBact4 | Firmicutes bacterium CAG:345 | CDD23257.1 | 0 | 3 |
| Cluster IV | FirBact5 | Firmicutes bacterium CAG:270 | CDD70844.1 | 1 | 3 |
| Cluster IV | FirBact6 | Firmicutes bacterium CAG:270 | CDD70826.1 | 3 | 3 |
| Cluster IV | RumObe10 | Ruminococcus obeum CAG:39 | CDD87342.1 | 3 | 3 |
| Cluster IV | CloSpe46 | Clostridium sp. CAG:288 | CDE16467.1 | 0 | 3 |
| Cluster IV | RumSpe12 | Ruminococcus sp. CAG:90 | CDE32116.1 | 3 | 3 |
| Cluster IV | RumSpe13 | Ruminococcus sp. CAG:90 | CDE32132.1 | 1 | 3 |
| Cluster IV | RumSpe14 | Ruminococcus sp. CAG:353 | CDE80869.1 | 0 | 3 |
| Cluster IV | MegElsd6 | Megasphaera elsdenii CAG:570 | CDF04683.1 | 2 | 3 |
| Cluster IV | MncSpec2 | Methanoculleus sp. CAG:1088 | CDF30674.1 | 3 | 3 |
| Cluster IV | RsbSpec5 | Roseburia sp. CAG:182 | CDF42615.1 | 0 | 3 |
| Cluster IV | CloBot29 | Clostridium botulinum Af84 | EPS53510.1 | 2 | 3 |
| Cluster IV | CloSorde | Clostridium sordellii VPI 9048 | EPZ58109.1 | 1 | 3 |
| Cluster IV | CloDiff6 | Clostridium difficile CD160 | EQF25001.1 | 2 | 3 |
| Cluster IV | CloBifer | Clostridium bifermentans ATCC 638 | EQK42135.1 | 1 | 3 |
| Cluster IV | MlsTinda | Methanolobus tindarius DSM 2278 | ETA66931.1 | 3 | 3 |
| Cluster IV | EubNodat | Eubacterium nodatum ATCC 33099 | ETK00068.1 | 2 | 3 |

**a**, Name shown in the *nifH* database (Heller *et al*.)

**b**, Mismatch number required for covering the sequences were shown; "0", "1", and "2" and the others were shown as "3".


Heller, P., H.J. Tripp, K. Turk-Kubo, and J.P. Zehr. 2014. ARBitrator: a software pipeline for on-demand retrieval of auto-curated *nifH* sequences from GenBank. Bioinformatics 30:2883–2890.

Ludwig, W., O. Strunk, R. Westram, et al. 2004. ARB : a software environment for sequence data. Nucleic Acid Res. 32:1363–1371.

Rice, P., L. Longden, and A. Bleasby. 2000. The European Molecular Biology Open Software Suite EMBOSS: The European Molecular Biology Open Software Suite. Trends Genet. 16:276–277.

Zehr, J.P., B.D. Jenkins, S.M. Short, and G.F. Steward. 2003. Nitrogenase gene diversity and microbial community structure: a cross-system comparison. Environ. Microbiol. 5:539–554.

Table S4. A total number of reads and OTUs after the quality check (OTU at 97% cut off).

| Primer set for amplification | Samples | Without singletons[a] | | Shannon index | Evenness |
|---|---|---|---|---|---|
| | | Reads | OTUs | | |
| 515F/806R (16S rRNA) | pale-tan mats | 29,671 | 603 | 1.77 | 0.28 |
| | white streamers | 40,214 | 529 | 1.15 | 0.20 |
| | pale-tan streamers | 35,665 | 622 | 1.93 | 0.33 |
| | gray streamer | 44,028 | 642 | 2.39 | 0.41 |
| MehtaF/MehtaR (*nifH*) | pale-tan mats | 20,873 | 31 | 0.27 | 0.11 |
| | white streamers | 22,248 | 40 | 1.71 | 0.52 |
| | pale-tan streamers | 6,210 | 45 | 0.76 | 0.22 |
| | gray streamers | 5,655 | 44 | 0.87 | 0.23 |
| PolF/PolR (*nifH*) | pale-tan mats | 8,553 | 37 | 0.74 | 0.38 |
| | white streamers | 1,848 | 21 | 1.37 | 0.45 |
| | pale-tan streamers | 25,897 | 71 | 0.08 | 0.05 |
| | gray streamers | 4,688 | 44 | 0.48 | 0.22 |

**a**, A total number of reads and OTUs after the quality check (OTU at 97% cut off) and the removal of singletons of four samples. 16S rRNA amplicon sequence data of the pale-tan mats and streamers analyzed in our previous report were used after treatments removing singleton sequences (48).

Table S5. Relative abundance and closest relatives of OTUs of 16S rRNA gene amplicons (>0.2% of total reads at least in one of the communities). Uncultured/environmental sample sequences were shown as the closest relatives if the identity to known species is below 90%. "0.00%" shows <0.0045% of total reads.

| Phylum | Genera or cluster | OTUs | Relative abundance | | | | Closest relatives (Accession No.) | Identity (%) |
|---|---|---|---|---|---|---|---|---|
| | | | Mat | Streamer | | | | |
| | | | pale-tan[a] | white | pale-tan[a] | gray | | |
| *Acidobacteria* | *Chloracidobacterium* | denovo34541 | 0.00% | 0.00% | 0.34% | 0.00% | Uncultured Acidobacteria bacterium clone YNP_SBC_BP4_B26 (HM448257) | 96.4% |
| | | | | | | | *Stenotrophobacter roseus* strain Ac_15_C4 (NR_146022), *Stenotrophobacter namibiensis* strain Ac_17_F2 (NR_146021) | 88.1% |
| *Aquificae* | *Hydrogenobacter* | denovo48891 | 6.29% | 1.50% | 12.3% | 23.6% | *Thermocrinis ruber* strain DSM 23557 (CP007028) | 96.0% |
| | | denovo28584 | 0.14% | 0.16% | 0.01% | 4.79% | *Thermocrinis ruber* strain DSM 23557 (CP007028) | 95.3% |
| | | denovo15700 | 0.66% | 0.03% | 0.19% | 0.20% | *Hydrogenobacter subterraneus* HGP1 (NR_024729), Thermothrix azorensis strain TM (NR_104832) | 99.6% |
| | | denovo18902 | 0.34% | 0.00% | 0.39% | 0.09% | *Thermocrinis ruber* DSM 23557 (CP007028), *Thermocrinis ruber* OC 1/4 (NR_121741) | 94.1% |
| | | denovo33234 | 0.02% | 0.18% | 0.03% | 2.18% | *Thermocrinis ruber* strain DSM 23557 (CP007028) | 94.9% |
| | | denovo33392 | 0.02% | 0.07% | 0.02% | 0.74% | *Thermocrinis ruber* strain DSM 23557 (CP007028) | 94.8% |
| | | denovo29919 | 0.09% | 0.00% | 0.22% | 0.06% | *Thermocrinis jamiesonii* GBS1 (NR_145905), Hydrogenobacter subterraneus strain HGP1 (NR_024729) | 96.4% |
| | *Sulfurihydrogenibium* | denovo14971 | 72.9% | 78.9% | 54.8% | 23.1% | *Sulfurihydrogenibium azorense* Az-Fu1 (NR_102858), *Sulfurihydrogenibium kristjanssonii* I6628 (NR_042660) | 100.0% |
| | | denovo14492 | 1.31% | 0.50% | 0.41% | 0.17% | *Sulfurihydrogenibium azorense* Az-Fu1 (NR_102858) | 96.8% |
| | | denovo53671 | 0.26% | 0.07% | 0.06% | 0.02% | *Sulfurihydrogenibium azorense* Az-Fu1 (NR_102858) | 98.4% |
| | | denovo50041 | 0.25% | 0.12% | 0.08% | 0.02% | *Sulfurihydrogenibium azorense* Az-Fu1 (NR_102858) | 98.4% |
| | | denovo26756 | 0.23% | 0.03% | 0.03% | 0.01% | *Sulfurihydrogenibium azorense* Az-Fu1 (NR_102858) | 97.2% |
| *Armatimonadetes* | uncultured | denovo15330 | 0.00% | 0.00% | 2.69% | 0.14% | Uncultured *Armatimonadetes* bacterium, clone: ST-B09_1 (AB735180) | 100% |
| | | | | | | | *Pelotomaculum thermopropionicum* strain SI (NR_074685) | 87% |
| | | denovo36368 | 0.00% | 0.00% | 0.53% | 0.00% | Uncultured bacterium clone NKB_56_N2 (JF826973) | 100% |
| | | | | | | | *Carboxydothermus islandicus* strain SET IS-9 (NR_108577), *Thermoterrabacterium ferrireducens* (U76364) | 86.8% |
| *Chlorobi* | OPB56 | denovo54962 | 0.01% | 0.00% | 1.07% | 0.02% | Uncultured *Chlorobi* bacterium DNA, fosmid clone: JFF027_B02 (AP011715) | 99.6% |
| | | | | | | | *Rhodothermus clarus* (AB252420), *Rhodothermus marinus* (AY986798) | 88.4% |
| | BSV26 | denovo27638 | 0.05% | 0.07% | 0.04% | 0.70% | Uncultured bacterium clone JGI-3 (KP289244) | 97.2% |
| | | | | | | | *Natranaerobius thermophilus* strain JW/NM-WN-LF (NR_074181) | 86.5% |
| *Dictyoglomi* | *Dictyoglomus* | denovo10055 | 0.31% | 0.33% | 0.12% | 1.59% | *Dictyoglomus turgidum* DSM 6724 (CP001251) | 99.2% |
| *Firmicutes* | *Caldicellurosiruptor* | denovo9761 | 0.55% | 0.03% | 0.01% | 0.07% | *Caldicellulosiruptor lactoaceticus* 6A (NR_074772), *Caldicellulosiruptor kristjanssonii* I77R1B (NR_074768), *Caldicellulosiruptor acetigenus* X6B (NR_115321) | 100.0% |
| *Thermodesulfobacteria* | *Caldimicrobium* | denovo20616 | 1.20% | 8.69% | 0.22% | 21.4% | *Caldimicrobium rimae* DS (NR_044283) | 98.8% |
| | | denovo155 | 0.47% | 0.52% | 0.04% | 0.01% | *Caldimicrobium thiodismutans* (LC055107) | 100.0% |
| *Thermotogae* | *Fervidobacterium* | denovo34660 | 1.13% | 0.02% | 0.00% | 0.00% | *Fervidobacterium nodosum* Rt17-B1 (NR_074093) | 100.0% |
| | *Fervidobacterium* | denovo17765 | 0.07% | 1.89% | 0.10% | 6.28% | *Fervidobacterium pennivorans* strain DYC (CP011393) | 100.0% |
| | *Thermotoga* | denovo2268 | 0.01% | 0.00% | 0.00% | 3.37% | *Thermotoga caldifontis* strain AZM44c09 (NR_133903) | 100.0% |
| | EM3 | denovo45350 | 0.00% | 0.03% | 0.00% | 0.28% | Uncultured bacterium clone dongzy2tff9406 (KU450045) | 100.0% |
| | | denovo46213 | 0.00% | 0.00% | 1.25% | 0.00% | Uncultured *Bacteroidetes* bacterium clone HGM-U-2 (AB539622) | 98.0% |
| | | | | | | | *Actinomadura rayongensis* strain RY35-68 (NR_134688) | 82.7% |
| | | denovo31056 | 0.01% | 0.00% | 12.2% | 0.39% | Uncultured bacterium clone NKB_56_T2 (JF826975) | 77.1% |
| | | | | | | | *Actinomadura atramentaria* strain DSM 43919 (NR_114850) | 82.3% |
| | | | | | | | *Acidobacterium* sp. SCGC AAA240-K18 (HQ675612) | 81.3% |
| *Thermus* | *Thermus* | denovo18496 | 2.42% | 0.08% | 4.02% | 3.28% | *Thermus arciformis* TH92 (NR_116251) | 100.0% |
| uncultured division | BP4 | denovo56209 | 0.02% | 0.00% | 0.73% | 0.07% | Uncultured bacterium clone NKB_56_V 16S ribosomal RNA gene | 99.6% |
| | | | | | | | *Thermoanaerobacter cellulolyticus* IFO 14436 (L09183) | 81.7% |

**a**, 16S rRNA amplicon sequence data of the pale-tan mats and streamers analyzed in our previous report were used after treatments removing singleton sequences (48).

**Table S6**. Relative abundance and closest relatives of NifH-OTUs amplicon amplified by two primer sets, PolF/PolR and MehtaF/MehtaR (>0.1% of total reads at least in one of the communities). “0.00%” shows below 0.0048% sequence in total reads.

| Groups (major phylum in group) | OTUs | Closest relatives (Accession no.) (aa % identity) | Relative abundance in total reads | | | | | | | |
|---|---|---|---|---|---|---|---|---|---|---|
| | | | pale-tan mats | | white streamers | | pale-tan streamers | | gray streamers | |
| | | | Pol | Mehta | Pol | Mehta | Pol | Mehta | Pol | Mehta |
| A (*Aquificae*) | NifH-OTU A1 | *Thermocrinis albus* DSM 14484 (WP_012991466) (87.7%) | 0.00% | 0.00% | 0.22% | 0.00% | 0.00% | 0.00% | 0.00% | 0.00% |
| | NifH-OTU A2 | *Thermocrinis albus* DSM 14484 (WP_012991466) (90.7%) | 0.00% | 0.00% | 0.16% | 0.00% | 0.00% | 0.00% | 0.00% | 0.00% |
| | NifH-OTU A3 | *Thermocrinis albus* DSM 14484 (WP_012991466) (87.9%) | 0.00% | 0.00% | 1.19% | 0.00% | 0.01% | 0.00% | 0.00% | 0.00% |
| | NifH-OTU A4 | *Hydrogenobacter thermophilus* TK-6 (WP_012963773) (94.3%) | 0.00% | 0.00% | 1.68% | 0.00% | 0.12% | 0.00% | 0.00% | 0.00% |
| | NifH-OTU A5 | *Thermocrinis albus* DSM 14484 (WP_012991466) (94.7%) | 0.02% | 0.01% | 3.57% | 7.16% | 0.06% | 0.81% | 0.00% | 0.02% |
| | NifH-OTU A6 | *Thermocrinis albus* DSM 14484 (WP_012991466) (93.8%) | 0.00% | 0.00% | 0.70% | 0.15% | 0.02% | 0.00% | 0.02% | 0.00% |
| | NifH-OTU A7 | *Thermocrinis albus* DSM 14484 (WP_012991466) (95.6%) | 0.00% | 0.00% | 66.7% | 17.2% | 0.51% | 1.37% | 0.58% | 0.30% |
| | NifH-OTU A8 | *Hydrogenobacter thermophilus* TK-6 (WP_012963773) (95.3%) | 0.00% | 0.00% | 2.65% | 2.74% | 0.18% | 1.14% | 0.02% | 0.00% |
| | | *Thermocrinis albus* DSM 14484 (WP_012991466) (95.3%) | | | | | | | | |
| | NifH-OTU A9 | *Hydrogenobacter thermophilus* TK-6 (WP_012963773) (95.6%) | 0.00% | 0.00% | 0.22% | 0.65% | 0.02% | 0.02% | 0.00% | 0.00% |
| | | *Thermocrinis albus* DSM 14484 (WP_012991466) (95.6%) | | | | | | | | |
| | NifH-OTU A10 | *Hydrogenobacter thermophilus* TK-6 (WP_012963773) (97.4%) | 0.04% | 0.15% | 2.06% | 5.36% | 0.12% | 1.48% | 0.00% | 0.37% |
| | NifH-OTU A11 | *Hydrogenobacter thermophilus* TK-6 (WP_012963773) (94.7%) | 0.00% | 0.00% | 0.00% | 0.57% | 0.00% | 0.39% | 0.00% | 0.00% |
| | NifH-OTU A12 | *Hydrogenobacter thermophilus* TK-6 (WP_012963773) (96.5%) | 0.00% | 0.11% | 0.05% | 1.81% | 0.01% | 0.31% | 0.00% | 0.04% |
| | | *Thermocrinis albus* DSM 14484 (WP_012991466) (96.5%) | | | | | | | | |
| | NifH-OTU A13 | *Hydrogenobacter thermophilus* TK-6 (WP_012963773) (85.1%) | 0.00% | 0.01% | 0.00% | 0.00% | 0.00% | 0.23% | 0.00% | 0.07% |
| | | *Thermocrinis albus* DSM 14484 (WP_012991466) (85.1%) | | | | | | | | |
| | NifH-OTU A14 | *Hydrogenobacter thermophilus* TK-6 (WP_012963773) (83.0%) | 0.00% | 0.02% | 0.00% | 0.00% | 0.00% | 0.23% | 0.00% | 0.04% |
| | | *Thermocrinis albus* DSM 14484 (WP_012991466) (83.0%) | | | | | | | | |
| | NifH-OTU A15 | *Thermocrinis albus* DSM 14484 (WP_012991466) (85.0%) | 0.08% | 0.00% | 0.00% | 0.00% | 0.26% | 0.00% | 0.30% | 0.00% |
| | NifH-OTU A16 | *Hydrogenobacter thermophilus* TK-6 (WP_012963773) (84.0%) | 0.00% | 0.00% | 0.00% | 0.00% | 0.01% | 0.24% | 0.00% | 0.07% |
| | | *Thermocrinis albus* DSM 14484 (WP_012991466) (84.0%) | | | | | | | | |
| | NifH-OTU A17 | *Thermocrinis albus* DSM 14484 (WP_012991466) (87.2%) | 0.00% | 0.01% | 0.00% | 0.00% | 0.00% | 0.14% | 0.00% | 0.09% |
| | NifH-OTU A18 | *Thermocrinis albus* DSM 14484 (WP_012991466) (85.1%) | 0.00% | 0.01% | 0.00% | 0.00% | 0.00% | 0.14% | 0.00% | 0.02% |
| | NifH-OTU A19 | *Thermocrinis albus* DSM 14484 (WP_012991466) (86.2%) | 0.04% | 0.00% | 0.00% | 0.00% | 0.11% | 0.00% | 0.04% | 0.00% |
| | NifH-OTU A20 | *Thermocrinis albus* DSM 14484 (WP_012991466) (90.0%) | 0.14% | 0.00% | 0.00% | 0.00% | 0.05% | 0.00% | 0.21% | 0.00% |
| | NifH-OTU A21 | *Thermocrinis albus* DSM 14484 (WP_012991466) (91.5%) | 39.89% | 3.47% | 1.30% | 0.29% | 97.5% | 84.0% | 84.9% | 22.0% |
| | NifH-OTU A22 | *Hydrogenobacter thermophilus* TK-6 (WP_012963773) (88.3%) | 0.00% | 0.02% | 0.00% | 0.00% | 0.00% | 0.14% | 0.00% | 0.04% |
| | NifH-OTU A23 | *Thermocrinis albus* DSM 14484 (WP_012991466) (87.9%) | 0.00% | 0.03% | 0.00% | 0.00% | 0.00% | 0.18% | 0.00% | 0.09% |
| | NifH-OTU A24 | *Thermocrinis albus* DSM 14484 (WP_012991466) (85.1%) | 0.00% | 0.00% | 0.00% | 0.00% | 0.00% | 0.14% | 0.00% | 0.02% |
| | NifH-OTU A25 | *Thermocrinis albus* DSM 14484 (WP_012991466) (93.4%) | 0.25% | 0.00% | 0.00% | 0.00% | 0.01% | 0.10% | 0.02% | 0.11% |
| B (*Cyanobacteria*) | NifH-OTU B1 | *Leptolyngbya boryana* (WP_017289029) (100%) | 0.00% | 0.00% | 0.00% | 0.00% | 0.00% | 0.00% | 0.00% | 0.27% |
| | NifH-OTU B2 | *Leptolyngbya* sp. CENA375 (AMQ98990) (99.1%) | 0.00% | 0.00% | 0.00% | 0.00% | 0.00% | 0.00% | 0.32% | 0.07% |
| | NifH-OTU B3 | *Leptolyngbya* sp. CENA375 (AMQ98990) (98.1%) | 0.00% | 0.00% | 0.11% | 0.00% | 0.00% | 0.00% | 0.00% | 0.00% |
| | NifH-OTU B4 | *Leptolyngbya boryana* IU 594 (AAA19031) (99.1%) | 0.00% | 0.00% | 0.00% | 0.00% | 0.00% | 0.00% | 0.06% | 0.19% |
| | NifH-OTU B5 | *Leptolyngbya* sp. CENA375 (AMQ98990) (98.1%) | 0.00% | 0.00% | 0.11% | 0.00% | 0.00% | 0.00% | 0.00% | 0.00% |
| | NifH-OTU B6 | *Leptolyngbya* sp. JSC-1 (ACO36768) (92.9%) | 0.00% | 0.00% | 0.00% | 0.00% | 0.00% | 0.00% | 0.00% | 0.11% |
| | NifH-OTU B7 | *Leptolyngbya* sp. CENA375 (AMQ98990) (99.1%) | 0.00% | 0.00% | 0.00% | 0.00% | 0.00% | 0.00% | 0.13% | 0.09% |
| | NifH-OTU B8 | *Oscillatoriales cyanobacterium* JSC-12 (WP_009769394) (92.0%) | 0.00% | 0.00% | 0.00% | 0.00% | 0.00% | 0.00% | 0.00% | 0.21% |
| | NifH-OTU B9 | *Fischerella ambigua* UTEX 1903 (AAC64640) (100%) | 0.00% | 0.00% | 0.00% | 0.00% | 0.00% | 0.00% | 0.11% | 0.04% |
| | | *Mastigocladus laminosus* Ind29 (AEX26862) (100%) | | | | | | | | |
| | NifH-OTU B10 | *Leptolyngbya* sp. KIOST-1 (WP_035984767) (100%) | 0.00% | 0.00% | 0.00% | 0.00% | 0.00% | 0.00% | 0.15% | 0.14% |
| | NifH-OTU B11 | *Lyngbya confervoides* (WP_039727379) (99.1%) | 0.00% | 0.00% | 0.00% | 0.00% | 0.00% | 0.00% | 0.13% | 1.15% |
| C | NifH-OTU C1 | *Rhizobium rosettiformans* W3 (ACT22748) (100%) | 0.11% | 0.00% | 0.05% | 0.00% | 0.02% | 0.00% | 0.06% | 0.00% |
| | | *Rheinheimera hassiensis* E48 (AGY46248) (100%) | | | | | | | | |
| | NifH-OTU C2 | *Wolinella succinogenes* DSM 1740 (WP_011139245) (89.0%) | 0.00% | 0.00% | 0.60% | 0.95% | 0.02% | 0.02% | 0.00% | 0.00% |
| | NifH-OTU C3 | *Azonexus hydrophilus* DSM 23864 (ABR29646) (99.1%) | 0.02% | 0.00% | 0.16% | 0.05% | 0.00% | 0.02% | 0.00% | 0.00% |
| | NifH-OTU C4 | *Azonexus hydrophilus* DSM 23864 (ABR29646) (98.1%) | 0.11% | 0.01% | 0.00% | 0.00% | 0.00% | 0.00% | 0.00% | 0.00% |
| | NifH-OTU C5 | *Azonexus hydrophilus* DSM 23864 (WP_028996168) (97.4%) | 0.04% | 0.01% | 0.70% | 0.28% | 0.01% | 0.08% | 0.00% | 0.00% |
| | | *Azovibrio restrictus* DSM 23866 (WP_026687661) (97.4%) | | | | | | | | |
| | NifH-OTU C6 | *Sedimenticola thiotaurini* SIP-G1 (WP_046860621) (97.4%) | 0.00% | 0.00% | 0.05% | 0.14% | 0.00% | 0.00% | 0.00% | 0.00% |
| D (*Nitrospirae*) | NifH-OTU D1 | *Thermodesulfovibrio* multispecies (WP_012546613) (96.5%) | 0.00% | 0.00% | 0.00% | 9.96% | 0.00% | 0.10% | 0.00% | 0.19% |
| | NifH-OTU D2 | *Thermodesulfovibrio* multispecies (WP_012546613) (86.0%) | 0.00% | 0.00% | 0.00% | 3.00% | 0.00% | 0.03% | 0.00% | 0.00% |
| | NifH-OTU D3 | Candidatus Magnetoovum chiemensis KJR40986 (91.2%) | 0.00% | 0.94% | 0.00% | 0.10% | 0.00% | 0.00% | 0.00% | 0.00% |
| | NifH-OTU D4 | *Thermodesulfovibrio aggregans* JCM 13213 (WP_059176981) (93.9%) | 0.08% | 0.06% | 0.32% | 0.00% | 0.03% | 0.02% | 0.06% | 0.04% |
| E (*Firmicutes*) | NifH-OTU E1 | *Caldicellulosiruptor hydrothermalis* 108 (WP_013403997) (94.0%) | 0.18% | 0.01% | 0.00% | 0.00% | 0.00% | 0.00% | 0.06% | 0.00% |
| | | *Caldicellulosiruptor kronotskyensis* 2002 (WP_013431038) (94.0%) | | | | | | | | |
| | | *Caldicellulosiruptor morganii* DSM 8990 (WP_045170428) (94.0%) | | | | | | | | |
| | NifH-OTU E2 | *Caldicellulosiruptor hydrothermalis* 108 (WP_013403997) (91.2%) | 0.00% | 0.00% | 0.32% | 0.00% | 0.01% | 0.00% | 0.00% | 0.00% |
| | | *Caldicellulosiruptor kronotskyensis* 2002 (WP_013431038) (91.2%) | | | | | | | | |
| | | *Caldicellulosiruptor morganii* DSM 8990 (WP_045170428) (91.2%) | | | | | | | | |
| | NifH-OTU E3 | *Caldicellulosiruptor hydrothermalis* 108 (WP_013403997) (100%) | 57.6% | 94.5% | 10.8% | 48.9% | 0.03% | 7.42% | 11.16% | 73.1% |
| | | *Caldicellulosiruptor kronotskyensis* 2002 (WP_013431038) (100%) | | | | | | | | |
| | NifH-OTU E4 | *Caldicellulosiruptor hydrothermalis* 108 (WP_013403997) (95.6%) | 0.02% | 0.08% | 0.27% | 0.06% | 0.01% | 0.00% | 0.00% | 0.02% |
| | | *Caldicellulosiruptor kronotskyensis* 2002 (WP_013431038) (95.6%) | | | | | | | | |
| | | *Caldicellulosiruptor morganii* DSM 8990 (WP_045170428) (95.6%) | | | | | | | | |
| | NifH-OTU E5 | *Caldicellulosiruptor hydrothermalis* 108 (WP_013403997) (93.8%) | 0.25% | 0.00% | 0.00% | 0.00% | 0.00% | 0.00% | 0.09% | 0.00% |
| | | *Caldicellulosiruptor kronotskyensis* 2002 (WP_013431038) (93.8%) | | | | | | | | |
| | | *Caldicellulosiruptor morganii* DSM 8990 (WP_045170428) (93.8%) | | | | | | | | |
| | NifH-OTU E6 | *Caldicellulosiruptor hydrothermalis* 108 (WP_013403997) (95.5%) | 0.21% | 0.00% | 6.22% | 0.01% | 0.11% | 0.00% | 0.06% | 0.00% |
| | | *Caldicellulosiruptor kronotskyensis* 2002 (WP_013431038) (95.5%) | | | | | | | | |
| | | *Caldicellulosiruptor morganii* DSM 8990 (WP_045170428) (95.5%) | | | | | | | | |
| F | NifH-OTU F1 | *Desulfotomaculum gibsoniae* DSM 7213 (WP_006523084) (94.6%) | 0.00% | 0.00% | 0.00% | 0.00% | 0.00% | 0.00% | 0.00% | 0.12% |
| | | *Desulfotomaculum alcoholivorax* DSM 16058 (WP_027364898) (94.6%) | | | | | | | | |
| | NifH-OTU F2 | Candidatus Margulisbacteria bacterium GWF2_35_9 (OGI10459) (86.9%) | 0.00% | 0.00% | 0.00% | 0.00% | 0.00% | 0.00% | 0.11% | 0.00% |
| G (*Chloroflexi*) | NifH-OTU G1 | *Roseiflexus castenholzii* DSM 13941 (WP_012122497) (100%) | 0.00% | 0.28% | 0.00% | 0.05% | 0.00% | 0.19% | 0.00% | 0.04% |
